# Evaluation and Future Challenges in a Self-Guided Web-Based Intervention With and Without Chat Support for Depression and Anxiety Symptoms During the COVID-19 Pandemic: Randomized Controlled Trial

**DOI:** 10.2196/53767

**Published:** 2024-09-30

**Authors:** Alejandro Dominguez-Rodriguez, Sergio Sanz-Gomez, Leivy Patricia González Ramírez, Paulina Erika Herdoiza-Arroyo, Lorena Edith Trevino Garcia, Anabel de la Rosa-Gómez, Joel Omar González-Cantero, Valeria Macias-Aguinaga, Paulina Arenas Landgrave, Sarah Margarita Chávez-Valdez

**Affiliations:** 1 Department of Psychology, Health and Technology University of Twente Enschede Netherlands; 2 Health Sciences Area Valencian International University Valencia Spain; 3 Universidad de Sevilla Seville Spain; 4 School of Medicine and Health Sciences Tecnologico de Monterrey Zapopan Mexico; 5 School of Psychology Universidad Internacional del Ecuador Quito Ecuador; 6 Faculty of Higher Studies Iztacala National Autonomous University of Mexico Mexico City Mexico; 7 Department of Behavioral Sciences Centro Universitario de los Valles Universidad de Guadalajara Guadalajara Mexico; 8 Faculty of Psychology National Autonomous University of Mexico Mexico City Mexico; 9 Escuela Libre de Psicología AC, ELPAC University of Behavioral Sciences Chihuahua Mexico; 10 Social Sciences Department Universidad Autónoma de Ciudad Juárez Ciudad Juárez Mexico

**Keywords:** self-guided web-based intervention, chat support, depression, anxiety, COVID-19, opinion, usability, randomized control trial

## Abstract

**Background:**

The COVID-19 pandemic has had an impact on mental health worldwide. Low- and middle-income countries were largely affected by it. Mexico was one of the most affected countries. Extended periods of lockdowns, isolation, and social distancing, among other factors, highlighted the need to introduce web-based psychological interventions to the Mexican population. In this context, Mental Health COVID-19 emerged as a self-guided web-based intervention (SGWI) aimed at adults to improve mental health during the COVID-19 pandemic.

**Objective:**

This study aims to assess the efficacy of 2 modalities of a self-guided intervention (with and without chat support) in reducing depression symptoms, generalized anxiety, community posttraumatic stress, widespread fear, anxiety, sleep quality, physiological and affective coping, and suicide ideation. In addition, it aimed to compare the moderating role of coping strategies, acceptance, and satisfaction in participants’ symptom reduction. We hypothesize that the self-guided, chat-supported modality will show higher efficacy than the modality without chat support in achieving clinical change and better performance as a moderator of depression symptoms, generalized anxiety, community posttraumatic stress, widespread fear, anxiety, sleep quality, physiological and affective coping, and suicide ideation, as well as an increase in participants’ satisfaction and acceptability.

**Methods:**

A randomized controlled trial was conducted. Data were collected from May 2020 to June 2022. We performed intrasubject measures at 4 evaluation periods: pretest, posttest, and follow-up measurements at 3 and 6 months. Differences between intervention groups were assessed through the Mann-Whitney *U* test for continuous variables and the chi-square test for categorical variables. Changes due to intervention were analyzed using Wilcoxon *W* test. Moderated regression analysis was performed to test the hypothesized moderating role of coping strategies, usability, and opinion about treatment on clinical change.

**Results:**

A total of 36 participants completed the intervention; of these, 5 (14%) were part of the SGWI group, and 31 (86%) were on the SGWI plus chat support (SGWI+C) group, which included a chat service with therapists. The perceived high complexity of the system for the SGWI group had a moderating effect associated with a lack of efficacy of the intervention regarding depression, but not when controlled for sociodemographic variables. A perception of lower helpfulness of the intervention was associated with poorer outcomes. Coping strategies did not show moderating effects.

**Conclusions:**

Enhancing the utility of web-based interventions for reducing clinical symptoms by incorporating a support chat to boost treatment adherence seemed to improve the perception of the intervention’s usefulness. Web-based interventions face several challenges, such as eliminating complexities in platform use and increasing the users’ perceived utility of the intervention, among other issues identified in the study.

**Trial Registration:**

ClinicalTrials.gov NCT04468893; https://clinicaltrials.gov/study/NCT04468893?tab=results

**International Registered Report Identifier (IRRID):**

RR2-10.2196/23117

## Introduction

### Background

On March 11, 2020, the World Health Organization declared COVID-19 a pandemic [1]. The COVID-19 pandemic has claimed the lives of >6 million people worldwide [2]. In response, governments worldwide introduced public health recommendations to reduce the transmission of the SARS-CoV-2 virus. Mobility restrictions and social isolation were the main measures introduced. These new conditions resulted in a lack of social interaction, social distancing, homeschooling for children, home offices for workers, and the closing of nonessential businesses [3,4]. Furthermore, stressors such as fear of infection, frustration, inadequate information, financial loss, stigma, separation from loved ones, loss of freedom, uncertainty about the progression of the disease, and feelings of helplessness were reported [5-7]. People experienced fear of contracting the disease or having their family members become ill or die [8], leading to decreased sleep quality and altered sleep patterns [9]. Due to these conditions, an increase in negative psychological effects was found [5], particularly regarding stress, depression, and anxiety, as well a heightened risk for developing posttraumatic stress disorder (PTSD) [10] and the increased risk of suicide. Thus, to mitigate the harms of COVID-19, there is a clear need to identify and promote effective psychological coping [11].

According to Kola et al [12], 83% of the world’s population lives in low-income and middle-income countries. These countries have been impacted not only by the government’s safety policies but also by the negative mental health sequelae of the COVID-19 pandemic. Mexico, a middle-income country, was one of the most affected countries during the COVID-19 pandemic [13,14], experiencing long periods of lockdown and ongoing uncertainty. Furthermore, symptomatology grew as the pandemic period increased [15]. People with noninfectious chronic diseases or COVID-19, as well as those who had to quarantine or be isolated from others, showed a higher risk of depression and anxiety than other population groups [16]. Moreover, while health systems were in acute crisis and struggling to provide prompt services, alternative web-based services delivering interventions were crucial during the pandemic [17].

Furthermore, positive psychology aims to enhance interventions that directly reduce depressive and anxious symptomatology. It is based on increasing positive variables and their mediating role in psychopathological problems. A systematic review and meta-analysis by Carr et al [18] evaluated the effects of 336 positive psychological interventions on increasing positive variables and decreasing psychopathological variables. These interventions included formats such as face-to-face, web-based, self-help, intervention groups, instruction from a therapist or coach, or bibliotherapy. The systematic review and meta-analysis identified that positive psychological interventions had a significant small to medium effect on well-being, strengths, quality of life, depression, anxiety, and stress. These positive effects were maintained at 3 months of follow-up. Similarly, based on a meta-analysis [19], positive psychological interventions were found to effectively improve subjective and psychological well-being and reduce depressive symptoms. Thus, while traditional psychotherapy has consistently shown efficacy in reducing psychopathological aspects, people show dissatisfaction in some parts of their lives [20]. Positive psychological interventions target the variables that explain dissatisfaction. Face-to-face psychological interventions, particularly those based on cognitive behavioral therapy (CBT), are effective in reducing symptoms of depression [21] and anxiety in the general population [22]. Furthermore, multimodal CBT has shown moderate effects in reducing anxiety and depression symptoms in primary care [23]. In addition, behavioral activation therapy (BAT) has shown improvement in depressive and anxious symptomatology, as well as an increase in activation [24] and social connectedness, with positive outcomes maintained at 1-year follow-up [25]. However, traditional face-to-face psychological interventions present some limitations, such as cost, difficulty of access, long waiting periods between requesting and receiving help, and the stigma that sometimes surrounds mental health [26,27]. Therefore, it has been suggested that future interventions should focus on developing platforms that can deliver safe digital mental health care treatment [28].

In this context, web-based psychological interventions, particularly those based on CBT, have been gaining strength and visibility in recent years as innovative and valuable tools aimed at promoting mental health [29], offering promising results in terms of efficacy and prevention strategies [30]. These interventions have shown to be as effective as in-person therapy [31,32] and provide solutions to the barriers of face-to-face treatments regarding flexibility, anonymity, low economic cost, ease of access, and the broad number of people who can potentially benefit from these mental health resources [26,33]. Furthermore, web-based interventions in a self-guided format allow users to progress through their treatment at their own pace, are cost-effective compared to in-person treatments, and allow privacy and anonymity [34].

Evidence suggests that internet-based treatments are effective for the treatment of anxiety disorders and depression [35,36]. Similarly, meta-analysis data reveal that these interventions, including face-to-face treatments, are also effective [36]. So, there is evidence that digital psychotherapeutic options are not inferior to their face-to-face counterparts [37]. It is noteworthy that self-guided programs enable greater dissemination and coverage of mental health services, contributing to innovative solutions and delivering the attention that users need. In addition, these programs can potentially reduce the rates of incidence and prevalence of psychological disorders. This study aims to contribute to the evidence of the effectiveness of self-management interventions for treating emotional distress in the context of extraordinary emergencies.

Moreover, positive psychology and internet-based interventions are 2 relatively young research fields. Nevertheless, there is evidence that it is beneficial for researchers and mental health professionals to consider delivering web-based positive psychology–based interventions [38]. Furthermore, users of internet interventions appreciate apps with various options, functionalities, and content, that is, high usability. Conversely, poor usability has emerged as the most common reason for abandoning mental health apps [39].

In this sense, we have designed, delivered, and evaluated Mental Health COVID-19 (in Spanish, Salud Mental COVID-19 [40]), a multicomponent, web-based self-administered intervention based on positive psychology, CBT, and BAT. This intervention aims at reducing symptoms of anxiety and depression and increasing sleep quality in the general Mexican population during and after the COVID-19 pandemic. Positive psychology, CBT, and BAT can be compatible as they work with thoughts, behaviors, and emotions, looking for the psychological well-being of people [41,42]. Given the consequences of the COVID-19 pandemic, this intervention intended to provide the Mexican general population with training in positive thinking, resilience, gratitude, interpersonal effectiveness skills, and problem-solving through the theory and techniques of these 3 approaches.

It should be noted that our intervention has a preventive approach, so according to what has been pointed out in various meta-analyses on programs to prevent depressive symptomatology, it is known that although the effect sizes are of small magnitude, the benefits are relevant [43-45].

### Objectives

This study had the following aims:

1. To evaluate the efficacy of the Mental Health COVID web-based intervention in reducing clinical symptoms of depression and anxiety among adult participants
2. To investigate the differential impact of the presence or absence of additional chat support on the efficacy of the intervention in reducing symptoms of depression and anxiety
3. To examine the potential moderating role of coping strategies, acceptance, and satisfaction on the clinical outcomes experienced by individuals participating in the intervention
4. To compare the acceptance and satisfaction levels between participants receiving the intervention with chat support and those receiving the intervention without chat support.

In addition, the discussion emphasizes the limitations and challenges detected during the implementation of the intervention, which is of great value for consideration in future similar proposals.

## Methods

### Hypotheses

To test the established objectives, we proposed the following hypotheses.

#### Primary Hypothesis

The self-guided web-based intervention plus chat support (SGWI+C) group will show major statistical changes in terms of efficacy than the self-guided web-based intervention (SGWI) group without chat support SGWI.

#### Secondary Hypotheses

The SGWI+C will show higher efficacy levels regarding clinical symptomatology, better performance regarding its moderating role of coping strategies, Generalized Anxiety Disorder (GAD), PTSD traits, anxiety and depression, and finally, the participants in the SGWI+C group will report higher rates of acceptance and satisfaction (positive opinion) than the web-based SGWI group.

### Study Design

A randomized controlled clinical trial with 2 independent groups was used, with intrasubject measures at 4 evaluation periods: pretest, posttest, and follow-ups at 3 and 6 months [46]. For this study, we followed the guidelines outlined in the CONSORT (Consolidated Standards of Reporting Trials) statement [47] and the CONSORT eHealth checklist [48].

Participants were randomly assigned to one of the following 2 groups:

1. Control active comparator: SGWI without assistance via chat
2. Experimental: SGWI+C provided by therapists in training and supervised by 2 authors of this study; the chat support was open for the users on the SGWI+C group to contact for any questions regarding the platform and the intervention

As described in Arenas-Landgrave et al [49], a personalized attention chat was available on the platform Salud Mental COVID, where the user could communicate with a mental health specialist upon entering the system. This staff was supervised by AdlR-G and PAL. ADR provided training to the therapists on using the chat service and monitored the proper use of this tool. The needs addressed in the chat could be emotional support, which refers to those occasions in which the person described symptoms of uncontrollable emotional discomfort; technical guidance about the platform or any of the modules; or referral to other sources of assistance in case of requiring more specialized care. When initiating contact with the patients, we conducted an assessment to explore the specific reasons individuals sought the service and the specific needs they required assistance with. This assessment aimed to address their concerns, provide guidance or resources as needed, and facilitate referrals to specialists if necessary [49]. For a further explanation of the chat and images, please refer to the study by Dominguez-Rodriguez et al [50].

### Participants and Eligibility Criteria

The intervention was delivered to the Mexican population. However, as it was an open web-based intervention, participants from other countries could also access the platform. The inclusion criteria were as follows: aged at least 18 years; voluntary participation; access to a technological device to receive the intervention, such as a computer, tablet, or mobile phone, and access to the internet; valid email address; and digital skills at an introductory level for using an operational system. The exclusion criteria were as follows: receiving psychological or pharmacological treatment during the study and not accepting the informed consent.

Eligibility criteria were assessed based on self-reported information provided by the participants before accessing the intervention platform.

### Recruitment Process

As we stated in the protocol article: “Participants will be recruited through advertisement in digital media (eg, notes in news magazines), as well as through dissemination on social networks” [50]. The participants were recruited through social networks. The primary social media platform used was Facebook, where a page called Salud Mental COVID (Mental Health COVID) was created for the project. On this Facebook page, there were shared advertisements for the study. Due to the relevance of the project, it had the support of the news media to be distributed to the general public during the initial phase of the pandemic. Some examples of the interviews conducted can be found in the study by Martínez-Prado [51] and Silerio [52]. The intervention started in May 2020, and the data were collected until June 2022. The reason for the long period of data collection is due to the COVID-19 restrictions and health measures that were still effective in Mexico. In addition, as in many developing countries, the vaccination was slower due to reduced vaccines available, and in the case of Mexico, the vaccination process was not equally distributed, with areas with higher vaccination percentages, such as Baja California Norte and Mexico City, compared with the rest of the country [53], and this could also have an impact on mental health. No economic or other types of incentives were provided to the participants, apart from the benefits provided by the free-of-cost intervention.

### Sample Size

The calculated sample size for this study was 166 participants (83 per group). Further study details are available in the protocol manuscript by Dominguez-Rodriguez et al [50]. The sample size was considered based on the effect sizes in controlled clinical studies in which the efficacy of web-based psychological interventions was evaluated. For this study, the Cohen *d* index was used, assuming that the variances of the 2 groups were homogeneous.

Furthermore, the study included 2 conditions: an a priori analysis to compare the means between the 2 independent groups and a conservative approach to include an effect size with an average magnitude of 0.25 (Cohen *d*, equivalent to Hedges *g*=0.5), a significance level (α) of .05 (*P*<.05, which corresponds to 95% CI), and a conventional statistical power of 80% (1–β=0.8). For the analysis, the software G*Power (version 3.1.6 [54]) was used, and a required sample size of 128 participants was obtained (64 per group).

However, the number of participants was increased by 30% to control the variable related to dropping out of participants during the treatment; this rate is reported in the literature on web-based treatments [55,56]. Thus, the total required sample size will be 166 participants (83 per group).

### Randomization

Once the evaluation was completed, the users were randomly assigned to one of the study conditions. The randomization was performed by an independent researcher using web-based randomization algorithm [57] at a ratio of 1:1 using the method of randomly permuted blocks. Due to a technical problem with the platform, at the beginning of the study, the users had only access to the intervention with chat, leaving this a higher weight on that group than the group without chat support. Once identified, it was corrected, and the distribution was ensured. The technical error of the system affected the difference in sample size in the intervention groups. However, this did not affect the equivalence between the groups in terms of preassessment indicators (eg, level of anxiety, depression, etc).

The participants were unaware that there was an intervention group and a comparison group, and they were unrelated.

### Instruments

#### Primary Outcome Measures

##### Beck Depression Inventory Second Version

The Beck Depression Inventory second version (BDI-II; [58]) is a widely used 21-item self-report inventory measuring the severity of depression in adolescents and adults consistent with the *Diagnostic and Statistical Manual of Mental Disorders, Fourth Edition* (*DSM-IV*), criteria for depression. The response options range from 0 to 3, except for items 16 and 18, which have 7 response options each. Total scores range from 0 to 63, where 0 to 13 points indicate minimal depression, 14 to 19 indicates mild depression, 20 to 28 indicates moderate depression, and 26 to 63 indicates severe depression. Studies of the psychometric properties of the Spanish version of the BDI-II for the Mexican population were conducted by Jurado et al [59] and González et al [60] for version II, showing adequate concurrent validity (*r*≥0.66) and reliability (Cronbach α values between 0.87 and 0.92) coefficients.

##### The GAD 7-Item Scale

This instrument is a screening tool for generalized anxiety. The GAD-7 [61] is a brief scale that consists of 7 items designed to measure the severity of symptoms of GAD. When screening for anxiety disorders, a score of ≥8 represents a reasonable cutoff point for identifying possible cases of GAD. Using a cutoff of 8, the GAD-7 reached good sensitivity and specificity. The maximum total score is 21. A score between 0 and 4 indicates that anxiety is not perceived, and a score between 15 and 21 shows perceived severe anxiety. Some items assess feeling nervous or anxious, inability to stop or control worrying, restlessness, and being easily annoyed. The questions in this scale are answered with scores ranging from 0 (never) to 3 (nearly every day). The version by Garcia-Campayo et al [62] was used for this study, which shows adequate concurrent validity (*r*≥0.70) and reliability (Cronbach α=0.93) coefficients.

##### Scale of Posttraumatic Stress Traits in Mexican Youth Exposed to Social Violence

The Scale of PTSD traits is a self-report scale developed in Colombia by Pineda Salazar et al [63], which comprises 5 domains related to PTSD symptomatology in correspondence with *Diagnostic and Statistical Manual of Mental Disorders, Fifth Edition* diagnosis criteria. The symptoms include having negative alterations in cognition and mood (criterion D), intrusion symptoms (criterion B), functional significance (criterion G), avoidance (criterion C), and arousal and reactivity alterations (criterion E). The scale rates the presence or absence of the abovementioned discrete categories. Chávez-Valdez et al [64] validated the scale to diagnose PTSD symptomatology based on *DSM-IV* criteria in Mexico. The scale consists of 24 items (eg, “Most of the time, I avoid the things and places that remind me of the situation”), with scores ranging from 1 (totally disagree) to 4 (totally agree). This is a discrete-categorical scale, with item loadings that range between 0.37 and 0.87. Correlations between factors ranged from *r*=0.45 to *r*=0.85, and good internal consistency of the Cronbach α between 0.92 and 0.97 [64] was found.

##### Widespread Fear Scale (Adapted in Northern Mexico)

This screening tool measures an emotional widespread fear such as fear of adversity in a particular context. It is composed of 7 items, with options of 0=nothing to 3=a lot.

In previous studies done by Ruiz Pérez [65] in Colombia, an acceptable internal consistency of 0.90 was reached. It consists of several items about the fear of being a victim of the context and 3 items about being afraid of the neighborhood or city. A highly significant and positive or directly proportional correlation was found between the Widespread Scale and the Social Insecurity Perception Scale (*r*=0.61; *P*<.001). In this way, there is convergent validity between the 2 scales. In turn, the divergent validity of the Social Insecurity Perception Scale was carried out because it contains a factor that measures the perception of citizen uncertainty through the widespread fear, which measures, in the opinion of the interviewer, the possibility of a perception of private uncertainties, which measures the opposite construct with an effect size (*r*=–0.28; *P*=.001). The adaptation of the instrument, performed in northern Mexico by Chávez-Valdez [66], obtained a Cronbach α coefficient of 0.92.

##### State-Trait Anxiety Inventory (Spanish Version)

The State-Trait Anxiety Inventory (STAI; Spanish version; [67]) instrument is a commonly used measure of trait and state anxiety. This inventory aims to distinguish between 2 types of anxiety. It can be used in clinical settings to diagnose anxiety and to distinguish it from depressive syndromes. This self-report measure categorizes symptoms related to anxiety as a personality trait (trait-anxiety) and distinguishes it from state-anxiety, which is defined as transitory anxiety that a person experiences at an anxious specific unforeseen event. It is composed of 40 items, 20 for the state and 20 for the trait. Internal consistency ranged from 0.86 to 0.95 [67].

##### The Pittsburgh Sleep Quality Index

The Pittsburgh Sleep Quality Index [68] is self-administered 19-item questionnaire that assesses sleep quality and disturbances during the past month. These items are rated considering the frequency or severity of sleep disturbance using scores ranging from 0 (not during the past month) to 3 (≥3 times a week). Items combine to form 7 components: sleep duration, sleep disturbance, sleep latency, daytime dysfunction, sleep efficiency, overall quality of sleep, and use of sleep medication [68]. The total score of the instrument is obtained by the sum of the component scores ranging from 0 to 21, with higher scores representing lower sleep quality. The evaluation of the Mexican population showed solid validity (*r*≥0.53) and reliability (α=.78) coefficients [69].

##### The Urban Insecurity Scale

The Urban Insecurity Scale [70], is a self-report measure, the Spanish version named “Un nuevo instrumento de evaluación psicológica: el Cuestionario de Inseguridad Urbana (CIU)” was originally developed by Vuanello [71]. This instrument has been designed based on a version of the Inventory of Anxiety Situations and Responses, a Spanish scale named Inventario de Situaciones y Respuestas de Ansiedad proposed by Miguel-Tobal and Cano-Vindel [72] in its first version since 1997. This scale comprises 15 items grouped into 4 dimensions characterized by reactions that indicate certain affective elements, such as worry, fear, feelings of insecurity, physiological activation, cognitive confrontation, and behavioral promotion components. The assessment of the test is made by adding the scores given by the person to each item of each scale. Thus, 4 scores are obtained: affective, cognitive, physiological, and behavioral, which represent the scores of each of the systems of answer. The total score is obtained by adding the 4 components previously described. For the interpretation of the profile, 4 levels of stress (or anxiety) have been defined: (1) absence of stress or normal stress, (2) moderate stress, (3) severe stress, and (4) extreme or posttraumatic stress. In the reliability analysis, the scale reached Cronbach α of 0.92 [70]. In this study, the α coefficient reached .91.

##### The Scale for Suicide Ideation

The Scale for Suicide Ideation [73] aims to assess the frequency of attitudes, behaviors, and plans to attempt suicide. It is divided into 19 items with response options of 0 to 2, giving a total score of 0 to 38, where a score ≥10 indicates an existing suicidal risk. This scale has been validated by González Macip et al [74] in the Mexican population, obtaining a Cronbach α of 0.84. For this study, only 2 items were applied to evaluate suicidal ideation: (1) for the past year, have you thought it would be better to be dead? and (2) have you ever tried to kill yourself?

#### Secondary Outcome Measures

##### Opinion About the Treatment

This questionnaire [75] comprises 4 questions that report the participants’ level of satisfaction with the treatment. The participants can report if they would recommend the treatment to a friend or family member, if they consider it helpful, and if they think that the treatment was difficult to manage or aversive. The questions are answered on a scale from 1 (nothing) to 10 (very much).

##### System Usability Scale

The System Usability Scale [76] is an instrument that has been designed to validate the usability of a system. It comprises 10 items, which are answered on a 5-point Likert-type scale concerning the degree of conformity of the product (1 completely disagree to 5 completely agree). All values must be added together and multiplied by 2.5 to obtain this scale’s global score, which ranges between 0 and 100. The System Usability Scale is a widely used standardized questionnaire, translated into many languages, such as Arabic, and Polish, among others [77]. Some items are “I think that I would like to use this system frequently,” and, “I needed to learn a lot of things before I could get going with this system.”

#### Intervention

The intervention Mental Health COVID-19 (ITLAB) comprises 15 modules, of which 11 (73%) are centered on positive psychology (eg, to provide tools to recognize personal abilities to recover after a stressful event), 2 on CBT (eg, the importance of emotions, and why they are experienced), and 2 on BAT (eg, performing a physical exercise that involves motor skills of the body). The intervention was delivered mainly in 2 formats: (1) videos that were uploaded on YouTube and embedded on the platform, and (2) PDF files that the participant could download that included further information about the session along with exercises and examples. The content of each video is presented in Table 1.

**Table 1:** Duration of each module.

Video	Duration
Module 1: understanding our emotions during the COVID-19 outbreak	12 minutes, 14 seconds
Module 2: reflection on preventive measures regarding COVID-19	9 minutes, 27 seconds
Module 3: time for gratitude	7 minutes, 13 seconds
Module 4: to the rhythm of life	13 minutes, 05 seconds
Module 5: resilience, facing adversity	14 minutes, 31 seconds
Module 6: helping my mind	9 minutes, 44 seconds
Module 7: taking control	18 minutes, 58 seconds
Module 8: smile and laugh	7 minutes, 41 seconds
Module 9: share concerns	7 minutes, 44 seconds
Module 10: separated but together	8 minutes, 12 seconds
Module 11: time to start	5 minutes, 23 seconds
Module 12: exercising my mind and body	7 minutes, 48 seconds
Module 13: spirituality	6 minutes, 49 seconds
Module 14: how to deal with grief over the loss of a loved one during the COVID-19 outbreak	11 minutes, 25 seconds
Module 15: my inner strength	15 minutes, 29 seconds

Similar contents and the chat function were used for the participants in the SGWI+C group. At the end of each module, the platform presented the participant with a 5-question survey with multiple-choice answers that evaluated the knowledge acquired in the module. The intervention was self-paced, meaning the participants could conclude the modules according to their time disposition.

### Data Analysis

Descriptive analysis of sample sociodemographic characteristics and clinical parameters at baseline were reported through the median and IQR for continuous variables to test for differences between 2 independent groups using nonparametric techniques, such as the Mann-Whitney *U* Test. Frequency and percentages were reported for categorical variables. Differences between intervention groups were assessed using Mann-Whitney *U* test for continuous variables and the chi-square test for categorical variables. Changes due to intervention between the pretest and posttest and 3- and 6-month follow-up assessments were analyzed using the Wilcoxon *W* test. They were applied to the SGWI+C group because the SGWI group did not answer the follow-up assessment invitations. As the postintervention assessment was only available to those who completed the entire treatment, all participants who had only accessed part of the modules were excluded from these analyses. Cases with missing values were excluded from each statistical test, so a maximum of n=31 was obtained for the pre- and postanalyses, n=18 in the 3-month follow-up, and n=4 in the 6-month follow-up. The size effect for the statistically significant differences obtained from the comparative analyses was estimated using Rosenthal *R* [78].

Moderating regression analysis was performed to test the hypothesized moderating role of coping strategies, usability, and opinion about treatment on clinical change. Only those variables showing statistically significant change were introduced as independent and dependent variables. Pretest scores of clinical variables were introduced as independent variables, posttest scores as dependent variables, and total scores of the Urban Insecurity Scale, System Usability Scale, and opinion questionnaire about treatment were introduced as moderating variables. In addition, sociodemographic variables (age, gender, and educational attainment) and the assignment to the SGWI or SGWI+C group were added to the model as control variables.

The model was built from 50,000 bootstrapping samples. The Johnson-Neyman interval was computed to identify the points on the slope at which there are significant changes in the effect of the moderator. All analyses were carried out using SPSS (version 26; IBM Corp). For moderating analysis, the macro PROCESS was used [79].

### Ethical Considerations

This study was approved by the ethics committee of the Escuela Libre de Psicología, Universidad de Ciencias del Comportamiento (ethics committee of the Free School of Psychology University of Behavioral Science) in Chihuahua, Mexico (reference number Folio 2008), and registered in ClinicalTrials.gov (NCT04468893), and in the International Registered Report Identifier (IRRID; DERR1-10.2196/23117). The participants provided their informed consent to participate in this study. The participants did not receive any compensation for participating in the study, besides receiving the invention totally free of charge.

## Results

### Overview

**Figure 1:**
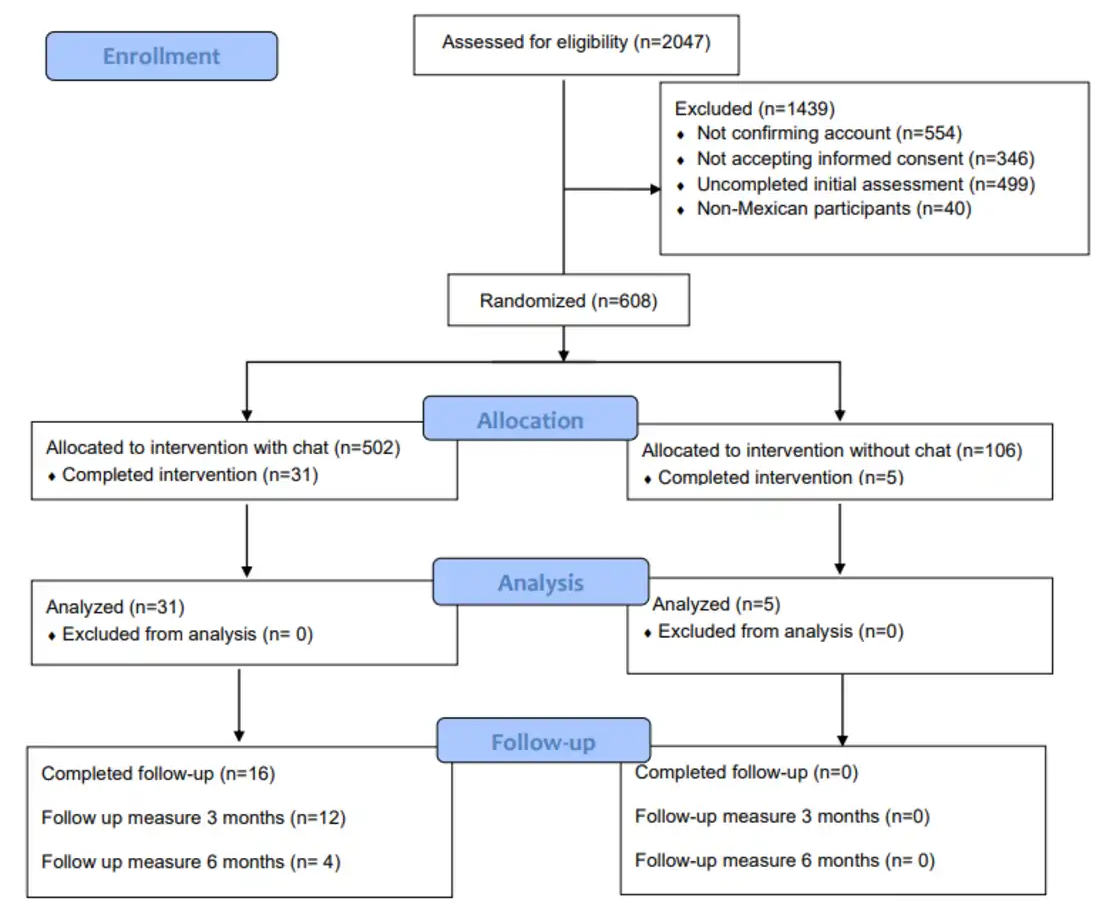
Flowchart of the study design.

In total, 2047 participants underwent the eligibility assessment. Of these, 1439 were excluded. Exclusion reasons were the following: not email account confirmation (554/1439, 38.5%), not accepting informed consent (346/1439, 24.04%), incomplete initial assessment (499/1439, 34.68%), and not from Mexico (40/1439, 2.78%). From the remaining 608 randomized participants to 1 of the 2 groups (Figure 1), 266 (43.7%) did not complete any module. Of the remaining participants, several dropped out during the intervention. At the start of the intervention, the main modules were where the participants dropped out. Table 2 presents this information in detail.

**Table 2:** Detailed dropout rates by the participants on intervention Salud Mental COVID.

Last module accessed	Participants (n=305)
1	145 (47.5)
2	35 (11.5)
3	18 (5.9)
4	16 (5.2)
5	7 (2.3)
6	8 (2.6)
7	4 (1.3)
8	5 (1.6)
9	4 (1.3)
10	1 (0.3)
11	4 (1.3)
12	0 (0)
13	1 (0.3)
14	5 (1.6)
15	52 (17); 16 (5.2) dropped out of the postintervention assessment)

### Sociodemographic Characteristics of the Sample

A total of 52 participants completed the intervention; however, 16 (31%) did not complete the postintervention assessment, so they were discarded from the analysis. Overall, 36 participants completed the intervention, including the pre- and postintervention assessment. Of these, 29 (81%) were female participants, and 7 (19%) were male participants. More than half of the participants (21/36, 58%) held a university degree, 7 (19%) had a master’s degree, 6 (17%) had high school studies, 1 (3%) had middle school studies, and 1 (3%) declared to have other academic attainments. Two-thirds (24/36, 67%) of the participants were working at the beginning of the intervention. The median age was 35 (IQR 37) years. Table 3 provides more details. Of the 36 participants who completed the intervention, 31 (86%) received the web-based SGWI+C assistance SGWI, whereas 5 (14%) participants received it without chat support (SGWI).

**Table 3:** Sociodemographic characteristics of the sample (N=36).

Characteristics			Participants, n (%)
**Gender**			
	Women	29 (81)	
	Men	7 (19)	
**Age (y)**			
	18-29	10 (28)	
	30-49	21 (58)	
	50-64	5 (14)	
**Education**			
	Secundaria (middle school)	1 (3)	
	Preparatoria (high school)	6 (17)	
	Bachelor’s	21 (58)	
	Master’s	7 (19)	
	Other (not specified)	1 (3)	
**Work**			
	Yes	24 (67)	
	No	12 (33)	
**Access to chat**			
	Yes	31 (86)	
	No	5 (14)	
**Mexican region**			
	Aguascalientes	1 (3)	
	Chihuahua	3 (8)	
	Ciudad de México	17 (47)	
	Estado de Mexico	11 (31)	
	Guanajuato	1 (3)	
	Hidalgo	1 (3)	
	Morelos	1 (3)	
	Oaxaca	1 (3)	

### Comparison of Clinical Symptoms Between Both Groups at the Pretest

Regarding clinical characteristics at baseline, the SGWI+C group scored mild depression more frequently (as per the BDI-II classification) than the SGWI group, in which minimal depression was the most common. This score stood out as a statistically significant difference between groups. No other differences were detected (Table 4).

**Table 4:** Clinical characteristics at baseline by intervention group.

Characteristics	SGWI^a^ (n=5)	SGWI+C^b^ (n=31)	*P* value^c^
BDI-II^d^, median (IQR)	2 (0.5-9)	14 (11-20)	.03
GAD-7^e^, median (IQR)	4 (1-5)	7 (4-12)	.07
The scale of Post-traumatic Stress Traits, median (IQR)	36 (32-36)	46 (31-64)	.26
Widespread fear scale, median (IQR)	18 (18-22)	18 (14-21)	.51
State anxiety, median (IQR)	38 (37-42)	45 (36-57)	.22
Trait anxiety, median (IQR)	37 (36-39)	42 (39-51)	.08
Pittsburgh Sleep Quality Index, median (IQR)	6 (2.5-10)	10 (7-13)	.13
Urban strategies coping strategies scale, median (IQR)	13 (13-15)	13 (11-17)	.49
Scale for suicide ideation	0 (0.0)	7 (23.3)	.56^f^

^a^SGWI: self-guided web-based intervention.

^b^SGWI+C: self-guided web-based intervention plus chat.

^c^*P* value for Mann-Whitney *U* test (bilateral).

^d^BDI-II: Beck Depression Inventory second version.

^e^GAD-7: Generalized Anxiety Disorder 7.

^f^*P* value for chi-square test (bilateral).

### Changes by Comparing Intervention Groups

Our primary hypothesis was to test whether the self-administered intervention with psychological assistance via chat would show more significant statistical gains in reducing anxiety and depression symptoms. For the SGWI group, symptom levels decreased compared to pre- and posttest. In contrast, for the SGWI+C group, the reduction in symptoms remained statistically relevant with minor to medium-sized effects for depression, widespread fear, and state anxiety, except for trait anxiety (Table 5).

**Table 5:** Changes between pre- and postintervention measures by intervention group.

Group			Preintervention measure		Postintervention measure		*P* value^a^		Rosenthal *R*
**BDI-II^b^**									
	SGWI^c^	2 (0.5-9)		1 (0,-3)		.10		—^d^	
	SGWI+C^e^	14 (11-20)		10 (4-10)		.01		−0.44	
**GAD-7^f^**									
	SGWI	4 (1-5)		1 (1-7)		.99		—	
	SGWI+C	7 (4-12)		6 (2-8)		.06		—	
**The scale of Post-traumatic Stress Traits**									
	SGWI	36 (32-36)		32 (30-48)		.69		—	
	SGWI+C	46 (31-64)		34 (26-46)		.06		—	
**Widespread Fear Scale**									
	SGWI	18 (18-22)		15 (14-16)		.34		—	
	SGWI+C	18 (14-21)		13 (12-18)		.01		−0.45	
**State anxiety**									
	SGWI	38 (37-42)		31 (28-37)		.5		—	
	SGWI+C	45 (36-57)		38 (33-49)		.05		−0.36	
**Trait anxiety**									
	SGWI	37 (36-39)		32 (31-42)		.5		—	
	SGWI+C	42 (39-51)		43.5 (34-47)		.05		—	
**Pittsburgh Sleep Quality Index**									
	SGWI	6 (2.5-10)		6 (3-10)		.71		—	
	SGWI+C	10 (7-13)		8 (5-11)		.09		—	
**Urban strategies coping strategies scale**									
	SGWI	13 (13-15)		16 (13-19)		.59		—	
	SGWI+C	13 (11-17)		12 (9-16)		.35		—	
Scale for suicide ideation									
	SGWI	0 (0.0)		0 (0.0)		—		—	
	SGWI+C	7 (23.3)		6 (20.0)		.56		—	

^a^*P* value for Wilcoxon signed ranks test.

^b^BDI-II: Beck Depression Inventory second version.

^c^SGWI: self-guided web-based intervention.

^d^Not available.

^e^SGWI+C: self-guided web-based intervention plus chat.

^f^GAD-7: Generalized Anxiety Disorder 7.

Posttraumatic symptoms, sleep disturbances, and suicidal ideation showed a reduction after the intervention for the entire group and the 2 groups separately. However, this decrease in symptomatology did not reach statistically significant levels.

### Moderating Variables

We hypothesized that coping strategies, acceptance, and satisfaction would function as moderating variables of clinical change in both intervention groups. Due to the low sample size of the SGWI group, moderating analyses were carried out for the whole sample. A series of moderating models were tested. Pre- and postintervention scores of variables with confirmed changes over intervention (BDI-II, Widespread Fear Scale, and State Anxiety through STAI) were introduced in the models as independent and dependent variables, respectively. Total scores of The Urban Insecurity Scale, System Usability Scale, and opinion questionnaire about treatment were introduced. As we failed to find the moderating effects in these models, we tested a second series of moderating regression for the BDI-II score, using individual items of the opinion questionnaire about treatment (Table 6) and the System Usability Scale (Table 7).

**Table 6:** Results on the opinion regarding the treatment.

Opinion questionnaire on treatment	Total (N=21)	SGWI^a^ (n=3)	SGWI+C^b^ (n=18)	*P* value
Satisfaction with intervention received, median (IQR; range)	9 (8-10; 5-10)	9 (7.5-10; 7-10)	9 (8-10; 5-10)	.96
Would recommend this intervention to a friend or family member, median (IQR; range)	10 (9-10; 5-10)	10 (9-10; 8-10)	10 (9-10; 5-10)	.66
Thinks that this intervention could be useful to treat other psychological problems, median (IQR; range)	9.5 (7-10; 1-10)	10 (8.5-10; 7-10)	9 (7-10; 1-10)	.36
Thinks the intervention has been useful in her case, median (IQR; range)	9 (7-10; 0-10)	9 (4-10; 0-10)	9 (7-10; 3-10)	.93
Found the intervention aversive or difficult to cope, median (IQR)	1 (0-9)	7.5 (2.5-10)	0 (0-7	.17

^a^SGWI: self-guided web-based intervention.

^b^SGWI+C: self-guided web-based intervention plus chat.

**Table 7:** Results on the System Usability Scale by the participants regarding the platform.

System Usability Scale	Total (N=21)	SGWI^a^ (n=3)	SGWI+C^b^ (n=18)	*P* value
1. I think I would like to visit this system regularly, median (IQR; range)	4 (4-5; 1-5)	3 (3-4; 3-4)	5 (4-5; 1-5)	.06
2. I found the system unnecessarily complex, median (IQR; range)^c^	1 (1-2; 1-4)	3 (1-4; 1-4)	1 (1-1; 1-4)	.09
3. I thought it was easy to use the system, median (IQR; range)	4 (2-5; 1-5)	3 (1-4; 1-4)	4.5 (2-5; 1-5)	.25
4. I think I would need the support of an expert to go through the system, median (IQR; range)^c^	1 (1-1; 1-4)	3 (1-4; 1-4)	1 (1-1; 1-2)	.004
5. I found the various possibilities of the system quite well integrated, median (IQR; range)	5 (3-5; 1-5)	3 (3-5; 3-5)	5 (3-5; 1-5)	.51
6. I thought there was too much inconsistency in the system, median (IQR; range)^c^	1 (1-3; 1-4)	3 (1-4; 1-4)	1 (1-2; 1-4)	.13
7. I imagine that most people would learn very quickly to use the system, median (IQR; range)	5 (4-5; 1-5)	4 (3-5; 3-5)	5 (4-5; 1-5)	.51
8. I found the system to be very large as I went through it, median (IQR; range)^c^	1 (1-3; 1-5)	3 (3-4; 3-4)	1 (1-3; 1-5)	.08
9. I felt very confident in using the system, median (IQR; range)	5 (4-5; 1-5)	3 (1-4; 1-4)	5 (4-5; 1-5)	.01
10. I need to learn a lot of things before I can use the system, median (IQR; range)^c^	1 (1-2; 1-5)	3 (1-5; 1-5)	1 (1-2; 1-5)	.13

^a^SGWI: self-guided web-based intervention.

^b^SGWI+C: self-guided web-based intervention plus chat.

^c^Reversed items: 2,4,6,8,10.

We found that item 2 of the System Usability Scale, “I found the system unnecessarily complex,” had a moderating effect. It was associated with a lack of efficacy of the intervention regarding depression, which increased with reports of higher perceived complexity (β=1.324; SE 0.390; *P*=.003). However, this effect disappeared when controlling for age, gender, and educational attainment. Low scores on item 4 of the opinion questionnaire about treatment, “Thinks the intervention has been useful in his/her case,” was also associated with poorer treatment outcomes (β=0.799; SE 0.241; *P*=.005 at low scores compared to no effect at high scores: β=–0.093; SE 0.316; *P*=.77). This moderation effect was independent of age, gender, educational attainment, and the SGWI or SGWI+C condition assignment. According to the Johnson-Neyman test [80], for those participants with a score above 7.6, the treatment would have typical efficacy on depression. However, participants below this score would be more resistant to treatment.

### Follow-Up Assessment

The follow-up assessment was unavailable for the SGWI group as they did not complete the 3-month or 6-month follow-ups. As for the participants in the SGWI+C group, although overall decreases were observed in the BDI-II and STAI’s State Anxiety, they lack statistical relevance. The Widespread Fear Scale maintained a statistically significant decrease at the 3-month follow-up (Rosenthal *R*=.63). However, the decrease was not maintained in the 6-month evaluation (Table 8).

**Table 8:** Results of the 3 and 6-month follow-up assessment for the self-guided web-based intervention plus chat group.

			BDI^a^ score		WFS^b^ score		STAI^c^ score
**Preintervention assessment**							
	n (%)	29 (100)		31 (100)		30 (100)	
	Median (IQR)	14 (11-20)		18 (14-21)		45 (36-57)	
**Postintervention assessment**							
	n (%)	29 (100)		31 (100)		30 (100)	
	Median (IQR)	10 (4-14)		13 (12-18)		38 (33-49)	
**3-month follow-up**							
	n (%)	12 (41)		12 (39)		12 (40)	
	Median (IQR)	8 (4-11.5)		13.5 (11.5-16.5)		36 (29-55.5)	
	*P* value^d^	.21		*.03*		.50	
**6-month follow-up**							
	n (%)	4 (14)		4 (13)		4 (13)	
	Median (IQR)	8.5 (1.5-15.5)		16 (12.5-17.5)		34.5 (20-51)	
	*P* value^e^	.85		.99		.99	

^a^BDI: Beck Depression Inventory.

^b^WFS: Widespread Fear Scale.

^c^STAI: State-Trait Anxiety Inventory.

^d^*P* value for Wilcoxon signed-ranks test between preintervention measure and 3-month follow-up.

^e^*P* value for Wilcoxon signed-ranks test between preintervention measure and 6-month follow-up.

### Acceptance and Satisfaction

Among our fourth hypothesis, we tested whether the participants in the SGWI+C group reported higher rates of acceptance and satisfaction compared to the SGWI group. There were no differences regarding satisfaction (Table 6). However, the SGWI+C group scored more positively than the other group’s 2 items on the System Usability Scale. Those items addressed autonomy when using the system (item 4) and confidence when using the system (item 9). No other differences emerged (Table 7).

## Discussion

### Principal Findings

The results of this study show a statistically significant change in depression (BDI-II), generalized fear, and anxiety (STAI, both state and trait scales) from the pretest to posttest in the total sample. However, changes in the generalized anxiety (GAD-7) were not identified. Thus, the results of this study could suggest modest results of the intervention with a small sample size, compared to other studies involving web-based interventions implemented during the pandemic with larger sample sizes and reduction in symptoms of anxiety [81-84] and depression [81,82,84].

According to the primary hypothesis of this study, the SGWI+C group showed statistically significant changes with minor to moderate effect sizes from the pretest to posttest in depression (BDI-II), generalized fear, and state anxiety (STAI-state). The SGWI group showed no statistically significant changes in the same measurements, although it should be noted that the SGWI+C group had higher BDI levels at baseline. In contrast, no changes in trait anxiety (STAI-trait) were observed in either group.

Some interventions have reported that psychological treatments without therapeutic guidance have had favorable results in variables such as depression and anxiety [82,84] or only in anxiety [83]. This contrasts with the results of this study where the SGWI group did not manage to reduce the levels of depression and anxiety. However, these studies did not consider a follow-up period or a randomized control group. Concerning fear regarding COVID-19, the study by Wahlund et al [85], which is also an intervention without therapeutic guidance, showed favorable results in this variable, in contrast to the SGWI group, in which a positive effect was not achieved. However, it should be noted that the study by Wahlund et al [85] did not have an active control group.

Regarding our secondary hypothesis, no significant differences in satisfaction were observed between the SGWI and the SGWI+C groups, and no moderating effects of coping strategies were identified either. This is consistent with the finding that internet-based interventions, with or without assistance, can have favorable effects [81].

In contrast, as part of the secondary hypothesis, it stands out that within acceptance, item 2 of the System Usability Scale (“I found the system unnecessarily complex”) had a moderating effect on depression levels. Besides, concerning satisfaction, lower scores in item 4 of the Treatment Opinion Questionnaire (“You think the intervention has been useful in your case”) were also associated with worse treatment outcomes.

The foregoing is consistent with the evidence identified in the study by Hanano et al [86] conducted to evaluate clinical outcomes in anxiety and depression in terms of treatment adherence. The authors in the study by Wei et al [84] pointed out that the user profile is important, therefore it was considered in this study by evaluating the acceptance, satisfaction, and usability of the tool. In addition, concerning the secondary hypothesis, it was not possible to evaluate whether the changes were maintained for the SGWI group because none of them completed the follow-up at 3 or 6 months. Regarding the participants in the SGWI+C group, no statistically significant data were observed to support the efficacy of the results at follow-up. Notably, only the favorable effect on generalized fear maintained a statistically significant decrease at a 3-month follow-up, which did not prevail at 6 months.

### Strengths

Regarding the strengths of this study, it was considered a follow-up at 3 and 6 months, in contrast to some studies that did not include a follow-up period [81-85]. Nevertheless, it was only possible to evaluate the follow-up in the SGWI+C group, whose results for generalized fear show that the favorable effects were maintained at 3 months and not at 6 months. Therefore, the replication of this study needs to be considered with caution. Future research can investigate the possible factors involved in the lack of long-term effects. Another strength of this study is the inclusion of variables considered mental health stressors, thus expanding from only psychopathological aspects [82]. Although we did not find any statistically significant associations between sleep quality and depression or anxiety, in a recent study by Coiro et al [87], not only did participants report high rates of anxiety and depression during the COVID-19 pandemic but they also reported poor sleep quality. The authors found a statistically significant association (*P*<.05) between COVID-19–related stressors (namely sleep quality, depression, and anxiety). Therefore, sleep quality may predict mental health situations [88]. Thus, including a sleep quality assessment and coping strategies as moderators of change could explain the phenomenon.

A further strength is that this study was conducted with Mexican participants, part of the Latin American population, where other authors have warned about the scarcity of web-based treatments. Although they imply the benefits, few studies have been published, including web-based interventions, and most are not randomized controlled trials [89]. Therefore, this study provides evidence to reduce this gap in web-based treatments that are targeted at developing countries that lack these types of interventions, compared to, for example, developed countries.

### Limitations and Future Challenges

The study has several limitations and has identified relevant elements that can be prevented or addressed to improve future similar projects. The main limitation is the small sample size for the SGWI group. Therefore, the planned analysis was modified to evaluate the second hypothesis regarding the moderating role of coping strategies, acceptance, and satisfaction variables on clinical change by group. Initially, this analysis was intended to be done separately for each group (SGWI and SGWI+C), but instead, it was performed considering the total sample. Furthermore, it was not possible to assess whether the changes were maintained for the SGWI group, as none completed follow-up at 3 or 6 months.

The inclusion of a therapist should be considered for future applications, as this feature has been reported to generate better results in internet-based psychological interventions than self-administered ones [81,90]. However, cost-benefit should be evaluated. As shown by Ruwaard et al [91], a decade of research on internet-based treatments across 9 randomized controlled trials has demonstrated that web-based therapist-assisted CBT has provided evidence supporting its effectiveness and efficacy of its application with outcomes comparable to in-person clinical practices, including high adherence rates.

A further limitation is the sample characteristics, which only comprise participants who accessed the Mental Health COVID-19 platform seeking treatment. This could lead to biases, as the group included people with access to technology who were actively seeking psychological help. This situation prevents generalizing the findings to the Mexican population, as it is a nonprobabilistic convenience sampling. Another limitation was the significant difference in depressive symptoms at baseline between the participants of both groups, which prevented adequate comparison before entering the study conditions. Caution should be taken in conclusions about this clinical variable. In subsequent studies, it would be relevant to assess the potential repercussions of this condition regarding treatment evolution, adherence, and dropout rates.

There was another possible bias in the sample. There were more women than men (29/36, 81% vs 7/36, 19%) and more participants with university studies (28/36, 78%) than other lower educational levels. Other web-based intervention studies have shown similar characteristics regarding gender and educational attainment [92], highlighting the importance of developing future treatments aimed at men and people with lower educational backgrounds. Furthermore, it is advisable to explore strategies such as establishing collaborations with public centers or corporations to facilitate access to this population group.

Another study limitation is the small sample size of the people who completed the intervention (n=36). Future studies should increase the number of users, for example, reviewing the recruitment criteria and interviewing potential participants to explore dropout reasons, barriers, and possible stigmas surrounding mental health. Web-based interventions often face difficulties such as medium to high dropout rates [92] and low adherence to treatment [93]. In this study, it was observed that the group that had chat support was able to complete the follow-up at 6 months, compared to those without therapist guidance. Having contact with a professional could be an element that helps adherence to the program. Conversely, in the satisfaction evaluation, items related to the system’s complexity and the intervention’s utility were associated with worse treatment outcomes. These elements could have contributed to the lack of adherence in some participants. Implementing pilot tests of the platforms in the general population to detect such elements would facilitate the success of the web-based intervention. Furthermore, this intervention included 15 sessions. Future studies should evaluate the adherence and effectiveness of shorter interventions. Similarly, it would be relevant for future studies to include measurement instruments with recent adaptations and validations carried out with the target population. These instruments with updated psychometric properties will reduce possible biases related to the measurement periods.

Moreover, in low- and middle-income countries, although mental health care is needed, it is not easily available. Web-based interventions could help reduce this gap [94]. For instance, Wang et al [95] found that self-help web-based programs can aid people who have experienced traumatic events. The participants in their study those who belonged to the rural group did not have easy internet access and were supported with web use throughout the process. This resulted in a positive adherence to the program. However, the authors speculate that their results could have been related to the face-to-face contact between the participants and the volunteers who helped with the internet service problems. Furthermore, a study conducted by Benjet et al [96] in Colombia and Mexico with a large sample size of 1319 university students, comparing internet-based CBT, self-guided internet-based CBT, and treatment as usual, obtained significant reductions in anxiety and depression.

In addition, another situation to consider is that web-based psychological interventions, particularly those of a self-guided type, remain relatively unknown in Latin American countries because these interventions are often offered to the population as therapeutic alternatives for a short time. Furthermore, few web-based interventions have been delivered in Mexico, free of charge for the user (eg, Grief COVID; [97]). Privacy and security of the users’ data should be explicitly warranted to increase trust among potential users of web-based resources [98]. These circumstances could have influenced the low participation that was evidenced in this study, and they should be considered in the elaboration of future web-based interventions.

An additional limitation is the small number of participants who completed the intervention in the SGWI group (n=5) compared to those from the SGWI+C group (n=31). This situation prevented comparing the intervention’s effectiveness in each group separately, which would have contributed to the study. Nevertheless, these results emphasize the importance of including elements such as chat in SGWIs that establish more direct and personalized contact with users. This interaction element was probably crucial to achieving greater adherence to treatment. Other studies that have used chat [99] as a tool for web-based psychologically guided interventions have shown that these resources have promoted the permanence of users and have been positively valued by them. The results of this study also show this trend identified in several of the works mentioned above highlighting the role of interactive elements such as chat, which act as resources that could encourage permanence in the intervention. Moreover, 56% of the participants did not complete the intervention, 16% more than estimated in the sample size calculation, which represents a high rate of dropouts. However, this rate is reported in the literature on web-based treatments [55,56] and could be explained by the same condition of emotional discomfort of the participants, the lack of private and adequate space for carrying out their intervention in the context of confinement due to the COVID-19 pandemic. Future analysis could be conducted with the current data collected to analyze the variables that could predict a higher likelihood of dropping out, such as the use of machine learning, a tool increasingly used in diverse studies to predict dropout [100] or completion [101] in web-based interventions, among other uses.

Another limitation of the study was the computer errors faced during the study. Regarding the randomization error, the platform used Tawk.io, which is a chat software designed to create live communication between users and the applications team. At first, the basic integration was built over the web on the client side, but this caused the problem of not being able to determine the user who was logged into the platform making it impossible to identify if it was assigned to the correct classification, leading to more participants receiving the chat assignment. This technical issue was resolved when the implementation of the Tawk.io plugin was transferred to server-side code; in this context, we do have a clear identification of user logged and correct assignment of the chat classification.

Furthermore, the Positive Psychological Functioning scale [102] was planned to be included in this paper. However, it could not be included because while the postmeasurements could be retrieved, there were electronic issues during the premeasurements.

Finally, psychotic disorder was proposed as an exclusion criterion in the study protocol. Nevertheless, we did not use this criterion when we conducted the study. We mention this inconsistency as a limitation of this research. Further interventions aimed at the general population should explore the presence of psychotic symptoms.

### Conclusions

The COVID-19 pandemic has impacted mental health worldwide. New ways of providing psychological help have highlighted the relevance of web-based interventions. Mental Health COVID-19 is a self-guided web-based treatment that has been applied to 36 Mexican participants to reduce depressive and anxious symptoms in the adult population. The results indicated a decrease in symptomatology, particularly in the group of participants who also received complementary support through chat, compared to the group that did not receive this assistance. This platform is a useful tool for the mental health care of the Mexican population that offers usability and easy access through interacting with videos, audio, and chat. It also contributes to increasing randomized controlled trials in the Latin American population.

Some challenges and recommendations for future SGWI are as follows: (1) to explore sampling strategies that allow a heterogeneous sample and reduce dropout during the early phases of the study; (2) to care inclusion and exclusion criteria to consider individuals with psychopathology or specific personality traits that may need a different approach; (3) to conduct pilot test to identify elements that could be complex for users, as well as their perceptions of the SGWI’s effectiveness; such pilot test would also help identify and address potential technical errors in the platform; (4) to consider the inclusion of interactive elements such as a chat or the involvement of therapist to accompany participants throughout the SGWI; (5) to seek strategies to facilitate long-term follow-up such as a personalized approach; and (6) to ensure the privacy and security of participant data to enhance trust in SGWI, mainly in developing countries.

## Appendix

Multimedia Appendix 1 CONSORT-EHEALTH checklist (V 1.6.1).

## Abbreviations

BAT: behavioral activation therapy
BDI-II: Beck Depression Inventory second version
CBT: cognitive behavioral therapy
CONSORT: Consolidated Standards of Reporting Trials
DSM-IV: Diagnostic and Statistical Manual of Mental Disorders, Fourth Edition
GAD: Generalized Anxiety Disorder
PTSD: posttraumatic stress disorder
SGWI: self-guided web-based intervention only
SGWI+C: self-guided web-based intervention plus chat support
STAI: State-Trait Anxiety Inventory

## Data Availability

The data sets generated during and analyzed during this study are available from the corresponding author on reasonable request.
