# Supplementary material for: Evaluation and Future Challenges in a Self-Guided Web-Based Intervention With and Without Chat Support for Depression and Anxiety Symptoms During the COVID-19 Pandemic: Randomized Controlled Trial
Source: JMIR Form Res. 2024 Sep 30;8:e53767. doi: 10.2196/53767 (PMC11474119; doi:10.2196/53767)
Supplement: Multimedia Appendix 1 [file formative_v8i1e53767_app1.pdf]

CONSORT-EHEALTH (V 1.6.1) - Submission/Publication Form

The CONSORT-EHEALTH checklist is intended for authors of randomized trials evaluating web-based and Internet-based applications/interventions, including mobile interventions, electronic games (incl multiplayer games), social media, certain telehealth applications, and other interactive and/or networked electronic applications. Some of the items (e.g. all subitems under item 5 - description of the intervention) may also be applicable for other study designs.

The goal of the CONSORT EHEALTH checklist and guideline is to be
a) a guide for reporting for authors of RCTs,
b) to form a basis for appraisal of an ehealth trial (in terms of validity)

CONSORT-EHEALTH items/subitems are MANDATORY reporting items for studies published in the Journal of Medical Internet Research and other journals / scientific societies endorsing the checklist.

Items numbered 1., 2., 3., 4a., 4b etc are original CONSORT or CONSORT-NPT (non-pharmacologic treatment) items.
Items with Roman numerals (i., ii, iii, iv etc.) are CONSORT-EHEALTH extensions/clarifications.

As the CONSORT-EHEALTH checklist is still considered in a formative stage, we would ask that you also RATE ON A SCALE OF 1-5 how important/useful you feel each item is FOR THE PURPOSE OF THE CHECKLIST and reporting guideline (optional).

Mandatory reporting items are marked with a red *.
In the textboxes, either copy & paste the relevant sections from your manuscript into this form - please include any quotes from your manuscript in QUOTATION MARKS,
or answer directly by providing additional information not in the manuscript, or elaborating on why the item was not relevant for this study.

YOUR ANSWERS WILL BE PUBLISHED AS A SUPPLEMENTARY FILE TO YOUR PUBLICATION IN JMIR AND ARE CONSIDERED PART OF YOUR PUBLICATION (IF ACCEPTED).
Please fill in these questions diligently. Information will not be copyedited, so please use proper spelling and grammar, use correct capitalization, and avoid abbreviations.

DO NOT FORGET TO SAVE AS PDF _AND_ CLICK THE SUBMIT BUTTON SO YOUR ANSWERS ARE IN OUR DATABASE !!!

Citation Suggestion (if you append the pdf as Appendix we suggest to cite this paper in the caption):
Eysenbach G, CONSORT-EHEALTH Group
[illegible]

Your response is too large. Try shortening some answers.

[illegible] Med Internet Res 2011;13(4):e126

URL: http://www.jmir.org/2011/4/e126/
doi: 10.2196/jmir.1923
PMID: 22209829

alejandro.dom.rod@gmail.com Switch account

Not shared

Draft not saved

* Indicates required question

Your name *

First Last

Alejandro Dominguez Rodriguez

Primary Affiliation (short), City, Country *

University of Toronto, Toronto, Canada

University of Twente

Your e-mail address *

abc@gmail.com

a.dominguezrodriguez@utwente.nl

Title of your manuscript *

Provide the (draft) title of your manuscript.

Evaluation and Future Challenges in a Self-guided Online Intervention with and without Chat Support for Depression and Anxiety Symptoms during the COVID-19 Pandemic: A Randomized Control Trial

Your response is too large. Try shortening some answers.

Name of your App/Software/Intervention *

If there is a short and a long/alternate name, write the short name first and add the long name in brackets.

Online intervention during COVID with chat sup

Evaluated Version (if any)

e.g. "V1", "Release 2017-03-01", "Version 2.0.27913"

Your answer

Language(s) *

What language is the intervention/app in? If multiple languages are available, separate by comma (e.g. "English, French")

Spanish

URL of your Intervention Website or App

e.g. a direct link to the mobile app on app in appstore (itunes, Google Play), or URL of the website. If the intervention is a DVD or hardware, you can also link to an Amazon page.

https://www.saludmentalcovid.com/

URL of an image/screenshot (optional)

Your answer

Your response is too large. Try shortening some answers.

Accessibility *

Can an enduser access the intervention presently?

- (selected) access is free and open
- access only for special usergroups, not open
- access is open to everyone, but requires payment/subscription/in-app purchases
- app/intervention no longer accessible
- Other:

Primary Medical Indication/Disease/Condition *

e.g. "Stress", "Diabetes", or define the target group in brackets after the condition, e.g. "Autism (Parents of children with)", "Alzheimers (Informal Caregivers of)"

Depression, Anxiety

Primary Outcomes measured in trial *

comma-separated list of primary outcomes reported in the trial

Beck Depression Inventory second version (BD

Secondary/other outcomes

Are there any other outcomes the intervention is expected to affect?

Opinion about the treatment, System Usability Scale

Your response is too large. Try shortening some answers.

Recommended "Dose" *

What do the instructions for users say on how often the app should be used?

- (selected) Approximately Daily
- Approximately Weekly
- Approximately Monthly
- Approximately Yearly
- "as needed"
- Other:

Approx. Percentage of Users (starters) still using the app as recommended after 3 months *

- (selected) unknown / not evaluated
- 0-10%
- 11-20%
- 21-30%
- 31-40%
- 41-50%
- 51-60%
- 61-70%
- 71%-80%
- 81-90%
- 91-100%
- Other:

Your response is too large. Try shortening some answers.

Overall, was the app/intervention effective? *

- (selected) yes: all primary outcomes were significantly better in intervention group vs control
- partly: SOME primary outcomes were significantly better in intervention group vs control
- no statistically significant difference between control and intervention
- potentially harmful: control was significantly better than intervention in one or more outcomes
- inconclusive: more research is needed
- Other:

Article Preparation Status/Stage *

At which stage in your article preparation are you currently (at the time you fill in this form)

- not submitted yet - in early draft status
- not submitted yet - in late draft status, just before submission
- submitted to a journal but not reviewed yet
- submitted to a journal and after receiving initial reviewer comments
- (selected) submitted to a journal and accepted, but not published yet
- published
- Other:

Your response is too large. Try shortening some answers.

Journal *

If you already know where you will submit this paper (or if it is already submitted), please provide the journal name (if it is not JMIR, provide the journal name under "other")

- ○ not submitted yet / unclear where I will submit this
- ○ Journal of Medical Internet Research (JMIR)
- ○ JMIR mHealth and UHealth
- ○ JMIR Serious Games
- ○ JMIR Mental Health
- ○ JMIR Public Health
- ◉ JMIR Formative Research
- ○ Other JMIR sister journal
- ○ Other:

Is this a full powered effectiveness trial or a pilot/feasibility trial? *

- ○ Pilot/feasibility
- ◉ Fully powered

Manuscript tracking number *

If this is a JMIR submission, please provide the manuscript tracking number under "other" (The ms tracking number can be found in the submission acknowledgement email, or when you login as author in JMIR. If the paper is already published in JMIR, then the ms tracking number is the four-digit number at the end of the DOI, to be found at the bottom of each published article in JMIR)

- ○ no ms number (yet) / not (yet) submitted to / published in JMIR
- ◉ Other: JFR ms#53767

Your response is too large. Try shortening some answers.

TITLE AND ABSTRACT

1a) TITLE: Identification as a randomized trial in the title

1a) Does your paper address CONSORT item 1a? *

I.e does the title contain the phrase "Randomized Controlled Trial"? (if not, explain the reason under "other")

- (selected) yes
- Other:

1a-i) Identify the mode of delivery in the title

Identify the mode of delivery. Preferably use "web-based" and/or "mobile" and/or "electronic game" in the title. Avoid ambiguous terms like "online", "virtual", "interactive". Use "Internet-based" only if Intervention includes non-web-based Internet components (e.g. email), use "computer-based" or "electronic" only if offline products are used. Use "virtual" only in the context of "virtual reality" (3-D worlds). Use "online" only in the context of "online support groups". Complement or substitute product names with broader terms for the class of products (such as "mobile" or "smart phone" instead of "iphone"), especially if the application runs on different platforms.

	1	2	3	4	5	
subitem not at all important	○	○	○	○	●	essential

Clear selection

Your response is too large. Try shortening some answers.

Does your paper address subitem 1a-i? *

Copy and paste relevant sections from manuscript title (include quotes in quotation marks "like this" to indicate direct quotes from your manuscript), or elaborate on this item by providing additional information not in the ms, or briefly explain why the item is not applicable/relevant for your study

1) Control Active Comparator: Self-applied online intervention (SAOI) without assistance via chat, and
2) Experimental: Self-applied online intervention with chat support (SAOI+C) provided by therapists in training and supervised by 2 authors of this study. The chat support was open for the users on the SAOI+C group to contact for any questions regarding the platform and the intervention.

1a-ii) Non-web-based components or important co-interventions in title

Mention non-web-based components or important co-interventions in title, if any (e.g., “with telephone support”).

	1	2	3	4	5	
subitem not at all important	○	○	○	○	○	essential

Does your paper address subitem 1a-ii?

Copy and paste relevant sections from manuscript title (include quotes in quotation marks "like this" to indicate direct quotes from your manuscript), or elaborate on this item by providing additional information not in the ms, or briefly explain why the item is not applicable/relevant for your study

Your answer

Your response is too large. Try shortening some answers.

1a-iii) Primary condition or target group in the title

Mention primary condition or target group in the title, if any (e.g., “for children with Type I Diabetes”) Example: A Web-based and Mobile Intervention with Telephone Support for Children with Type I Diabetes: Randomized Controlled Trial

	1	2	3	4	5	
subitem not at all important	○	○	○	○	○	essential

Does your paper address subitem 1a-iii? *

Copy and paste relevant sections from manuscript title (include quotes in quotation marks "like this" to indicate direct quotes from your manuscript), or elaborate on this item by providing additional information not in the ms, or briefly explain why the item is not applicable/relevant for your study

The intervention was delivered to the Mexican population

1b) ABSTRACT: Structured summary of trial design, methods, results, and conclusions

NPT extension: Description of experimental treatment, comparator, care providers, centers, and blinding status.

1b-i) Key features/functionalities/components of the intervention and comparator in the METHODS section of the ABSTRACT

Mention key features/functionalities/components of the intervention and comparator in the abstract. If possible, also mention theories and principles used for designing the site. Keep in mind the needs of systematic reviewers and indexers by including important synonyms. (Note: Only report in the abstract what the main paper is reporting. If this information is missing from the main body of text, consider adding it)

	1	2	3	4	5	
subitem not at all important	○	○	○	○	◉	essential

Clear selection

Your response is too large. Try shortening some answers.

Does your paper address subitem 1b-i? *

Copy and paste relevant sections from the manuscript abstract (include quotes in quotation marks "like this" to indicate direct quotes from your manuscript), or elaborate on this item by providing additional information not in the ms, or briefly explain why the item is not applicable/relevant for your study

Therefore, this study aims 1) to assess the self-applied online intervention's efficacy in reducing anxiety and depression symptoms, 2) to identify the effectiveness related to the presence versus absence of additional chat support, 3) to explore the potential moderating role of coping strategies, acceptance, and satisfaction in clinical change, 4) Assess the outcomes regarding participant acceptance and satisfaction when comparing the intervention with chat support to the intervention without chat support. A randomized controlled trial was conducted. Data were collected from May 2020 to June 2022, including pretest, posttest, and follow-up measurements of 3 and 6 months

1b-ii) Level of human involvement in the METHODS section of the ABSTRACT

Clarify the level of human involvement in the abstract, e.g., use phrases like “fully automated” vs. “therapist/nurse/care provider/physician-assisted” (mention number and expertise of providers involved, if any). (Note: Only report in the abstract what the main paper is reporting. If this information is missing from the main body of text, consider adding it)

	1	2	3	4	5	
subitem not at all important	○	○	○	○	○	essential

Does your paper address subitem 1b-ii?

Copy and paste relevant sections from the manuscript abstract (include quotes in quotation marks "like this" to indicate direct quotes from your manuscript), or elaborate on this item by providing additional information not in the ms, or briefly explain why the item is not applicable/relevant for your study

Your answer

Your response is too large. Try shortening some answers.

1b-iii) Open vs. closed, web-based (self-assessment) vs. face-to-face assessments in the METHODS section of the ABSTRACT

Mention how participants were recruited (online vs. offline), e.g., from an open access website or from a clinic or a closed online user group (closed usergroup trial), and clarify if this was a purely web-based trial, or there were face-to-face components (as part of the intervention or for assessment). Clearly say if outcomes were self-assessed through questionnaires (as common in web-based trials). Note: In traditional offline trials, an open trial (open-label trial) is a type of clinical trial in which both the researchers and participants know which treatment is being administered. To avoid confusion, use “blinded” or “unblinded” to indicated the level of blinding instead of “open”, as “open” in web-based trials usually refers to “open access” (i.e. participants can self-enrol). (Note: Only report in the abstract what the main paper is reporting. If this information is missing from the main body of text, consider adding it)

	1	2	3	4	5	
subitem not at all important	○	○	○	○	○	essential

Does your paper address subitem 1b-iii?

Copy and paste relevant sections from the manuscript abstract (include quotes in quotation marks "like this" to indicate direct quotes from your manuscript), or elaborate on this item by providing additional information not in the ms, or briefly explain why the item is not applicable/relevant for your study

Your answer

1b-iv) RESULTS section in abstract must contain use data

Report number of participants enrolled/assessed in each group, the use/uptake of the intervention (e.g., attrition/adherence metrics, use over time, number of logins etc.), in addition to primary/secondary outcomes. (Note: Only report in the abstract what the main paper is reporting. If this information is missing from the main body of text, consider adding it)

	1	2	3	4	5	
subitem not at all important	○	○	○	○	◉	essential

Clear selection

Your response is too large. Try shortening some answers.

Does your paper address subitem 1b-iv?

Copy and paste relevant sections from the manuscript abstract (include quotes in quotation marks "like this" to indicate direct quotes from your manuscript), or elaborate on this item by providing additional information not in the ms, or briefly explain why the item is not applicable/relevant for your study

Data were collected from May 2020 to June 2022, including pretest, posttest, and follow-up measurements of 3 and 6 months. Thirty-six participants finished the intervention, 5 were part of the Self-Applied Online Intervention Group (SAOI), and 31 were on the Self-Applied Online Intervention Group+Chat (SAOI+C) intervention, including a chat service with therapists. The intervention was delivered through fifteen videos with a duration between 10 to 20 minutes, plus a PDF with review materials accessible to participants. The perceived high complexity of the system for the SAOI group had a moderating effect associated with a lack of efficacy of the intervention regarding depression, but not when controlled for sociodemographic variables. Perception of lower helpfulness of the intervention was associated with poorer outcomes. Coping strategies did not show moderating effects

1b-v) CONCLUSIONS/DISCUSSION in abstract for negative trials

Conclusions/Discussions in abstract for negative trials: Discuss the primary outcome - if the trial is negative (primary outcome not changed), and the intervention was not used, discuss whether negative results are attributable to lack of uptake and discuss reasons. (Note: Only report in the abstract what the main paper is reporting. If this information is missing from the main body of text, consider adding it)

	1	2	3	4	5	
subitem not at all important	○	○	○	○	◉	essential

Clear selection

Does your paper address subitem 1b-v?

Copy and paste relevant sections from the manuscript abstract (include quotes in quotation marks "like this" to indicate direct quotes from your manuscript), or elaborate on this item by providing additional information not in the ms, or briefly explain why the item is not applicable/relevant for your study

Relevance of online interventions aimed at reducing clinical symptoms and highlighting the contribution of adding a support chat as a tool to promote treatment adherence and a [illegible]

Your response is too large. Try shortening some answers.

INTRODUCTION

2a) In INTRODUCTION: Scientific background and explanation of rationale

2a-i) Problem and the type of system/solution

Describe the problem and the type of system/solution that is object of the study: intended as stand-alone intervention vs. incorporated in broader health care program? Intended for a particular patient population? Goals of the intervention, e.g., being more cost-effective to other interventions, replace or complement other solutions? (Note: Details about the intervention are provided in “Methods” under 5)

	1	2	3	4	5	
subitem not at all important	○	○	○	○	◉	essential

Clear selection

Your response is too large. Try shortening some answers.

Does your paper address subitem 2a-i? *

Copy and paste relevant sections from the manuscript (include quotes in quotation marks "like this" to indicate direct quotes from your manuscript), or elaborate on this item by providing additional information not in the ms, or briefly explain why the item is not applicable/relevant for your study

Positive psychology and Internet-based interventions are two relatively young research fields. Nevertheless, there has been evidence that it is helpful for researchers and mental health professionals to consider the benefits of delivering online positive psychology-based interventions [33]. Furthermore, there is evidence that internet intervention users appreciate apps with various options, functionalities, and content, i.e., high usability. On the other hand, poor usability emerged as the most common reason for abandoning mental health apps [34].

In this senseTherefore, we have designed, delivered and evaluated Mental Health COVID-19 (in Spanish, Salud Mental COVID-19) a multi-component web-based self-administered intervention, based on positive psychology, CBT, and BAT to reduce symptoms of anxiety and depression and to increase sleep quality in the general Mexican population during and after the COVID-19 pandemic. Positive psychology, CBT, and BAT can be compatible as they work with thoughts, behaviors, and emotions, looking for the psychological well-being of people [35, 36]. Given the consequences of the COVID-19 pandemic, this intervention intended to provide the general population with training in positive thinking, resilience, gratitude, interpersonal effectiveness skills, and problem-solving, through the theory and techniques of these three approaches.

2a-ii) Scientific background, rationale: What is known about the (type of) system

Scientific background, rationale: What is known about the (type of) system that is the object of the study (be sure to discuss the use of similar systems for other conditions/diagnoses, if appropiate), motivation for the study, i.e. what are the reasons for and what is the context for this specific study, from which stakeholder viewpoint is the study performed, potential impact of findings [2]. Briefly justify the choice of the comparator.

	1	2	3	4	5	
subitem not at all important	○	○	○	◉	○	essential

Clear selection

Your response is too large. Try shortening some answers.

Does your paper address subitem 2a-ii? *

Copy and paste relevant sections from the manuscript (include quotes in quotation marks "like this" to indicate direct quotes from your manuscript), or elaborate on this item by providing additional information not in the ms, or briefly explain why the item is not applicable/relevant for your study

The evidence suggests that Internet-based treatments are effective for the treatment of anxiety disorders and depression [30, 31]. Likewise, meta-analysis data reveal that these interventions, are so effective such as face-to-face treatments [31]. In particular, the treatments completely self-applied in mid technology have shown lower rates of improvement compared to those who have synchronous support with a therapist [32]. However, it stands out that self-applied programs enable greater dissemination and coverage of mental health services that contribute to provide innovative solutions and deliver to users attention that they need it and that, eventually, they will reduce the rates of incidence and prevalence of psychological disorders. The present study aims to contribute to the evidence of the effectiveness of self-management interventions for the treatment of emotional distress in a context of extraordinary emergency.

2b) In INTRODUCTION: Specific objectives or hypotheses

Does your paper address CONSORT subitem 2b? *

Copy and paste relevant sections from the manuscript (include quotes in quotation marks "like this" to indicate direct quotes from your manuscript), or elaborate on this item by providing additional information not in the ms, or briefly explain why the item is not applicable/relevant for your study

This study has four aims. First, to assess the efficacy of Mental Health COVID online intervention in reducing clinical symptoms of depression and anxiety in Mexican adults. Second, to identify the effectiveness related to the presence versus absence of additional chat support. Third, to explore the potential moderating role of coping strategies, acceptance, and satisfaction in clinical change and fourth to evaluate the results of acceptance and satisfaction of the participants receiving the intervention with chat support vs. the intervention without chat support.

METHODS

Your response is too large. Try shortening some answers.

Does your paper address CONSORT subitem 3a? *

Copy and paste relevant sections from the manuscript (include quotes in quotation marks "like this" to indicate direct quotes from your manuscript), or elaborate on this item by providing additional information not in the ms, or briefly explain why the item is not applicable/relevant for your study

A randomized controlled clinical superiority trial with two independent groups was used, with intrasubject measures at four evaluation periods: pretest, posttest, follow-up at 3 months, and follow-up at 6 months [37]. For this study, we followed the guidelines outlined in the Consolidated Standards of Reporting Trials (CONSORT) statement [38] and the CONSORT eHealth checklist [39].
Participants were randomly assigned to one of two groups:
1) Control Active Comparator: Self-applied online intervention (SAOI) without assistance via chat, and
2) Experimental: Self-applied online intervention with chat support (SAOI+C) provided by therapists in training and supervised by 2 authors of this study. The chat support was open for the users on the SAOI+C group to contact for any questions regarding the platform and the intervention.

3b) Important changes to methods after trial commencement (such as eligibility criteria), with reasons

Your response is too large. Try shortening some answers.

Does your paper address CONSORT subitem 3b? *

Copy and paste relevant sections from the manuscript (include quotes in quotation marks "like this" to indicate direct quotes from your manuscript), or elaborate on this item by providing additional information not in the ms, or briefly explain why the item is not applicable/relevant for your study

The study has some limitations. The main limitation is that due to the small sample size for the SAOI group, the planned analysis was modified, which corresponded to the evaluation of the second hypothesis on the moderating role of coping strategies, acceptance, and satisfaction variables on clinical change by group. Initially, it was intended to be done separately for each group (SAOI and SAOI+C), but instead, it was performed considering the total sample. Furthermore, it was not possible to assess whether the changes were maintained for the SAOI group, as none completed follow-up at 3 or 6 months.

Finally, psychotic disorder was proposed as an exclusion criterion in the study protocol. Nevertheless, we did not use this criterion when we conducted the study. We mention here this inconsistency as a limitation of this research. Further interventions aimed at the general population should explore the presence of psychotic symptoms.

3b-i) Bug fixes, Downtimes, Content Changes

Bug fixes, Downtimes, Content Changes: ehealth systems are often dynamic systems. A description of changes to methods therefore also includes important changes made on the intervention or comparator during the trial (e.g., major bug fixes or changes in the functionality or content) (5-iii) and other “unexpected events” that may have influenced study design such as staff changes, system failures/downtimes, etc. [2].

	1	2	3	4	5	
subitem not at all important	○	○	○	◉	○	essential

Clear selection

Your response is too large. Try shortening some answers.

Does your paper address subitem 3b-i?

Copy and paste relevant sections from the manuscript (include quotes in quotation marks "like this" to indicate direct quotes from your manuscript), or elaborate on this item by providing additional information not in the ms, or briefly explain why the item is not applicable/relevant for your study

Due to a technical problem with the platform, at the beginning of the study, the users had only access to the intervention with chat, leaving this a higher weight on that group than the group without chat support. Once identified, it was corrected, and the distribution was ensured. The technical error of the system affected the difference in sample size in the intervention groups. However, this did not affect the equivalence between the groups in terms of pre-assessment indicators (e.g. level of anxiety, depression, etc.).

Furthermore, the Positive Psychological Functioning scale [98] was planned to be included in this article. However, it could not be included because while the post-measurements could be retrieved, there were electronic issues during the pre-measurement.

4a) Eligibility criteria for participants

Does your paper address CONSORT subitem 4a? *

Copy and paste relevant sections from the manuscript (include quotes in quotation marks "like this" to indicate direct quotes from your manuscript), or elaborate on this item by providing additional information not in the ms, or briefly explain why the item is not applicable/relevant for your study

The inclusion criteria were:
1. To be 18 years old minimum and no age limit.
2. Voluntary participation.
3. Access to a technological device to receive the intervention, such as a computer, tablet, or mobile phone, and access to the internet.
4. Valid email address.
5. Digital skills at an introductory level for using an operational system.

Your response is too large. Try shortening some answers.

4a-i) Computer / Internet literacy

Computer / Internet literacy is often an implicit “de facto” eligibility criterion - this should be explicitly clarified.

	1	2	3	4	5	
subitem not at all important	○	○	◉	○	○	essential

Clear selection

Does your paper address subitem 4a-i?

Copy and paste relevant sections from the manuscript (include quotes in quotation marks "like this" to indicate direct quotes from your manuscript), or elaborate on this item by providing additional information not in the ms, or briefly explain why the item is not applicable/relevant for your study

Your answer

4a-ii) Open vs. closed, web-based vs. face-to-face assessments:

Open vs. closed, web-based vs. face-to-face assessments: Mention how participants were recruited (online vs. offline), e.g., from an open access website or from a clinic, and clarify if this was a purely web-based trial, or there were face-to-face components (as part of the intervention or for assessment), i.e., to what degree got the study team to know the participant. In online-only trials, clarify if participants were quasi-anonymous and whether having multiple identities was possible or whether technical or logistical measures (e.g., cookies, email confirmation, phone calls) were used to detect/prevent these.

	1	2	3	4	5	
subitem not at all important	○	○	◉	○	○	essential

Clear selection

Your response is too large. Try shortening some answers.

Does your paper address subitem 4a-ii? *

Copy and paste relevant sections from the manuscript (include quotes in quotation marks "like this" to indicate direct quotes from your manuscript), or elaborate on this item by providing additional information not in the ms, or briefly explain why the item is not applicable/relevant for your study

“Participants will be recruited through advertisement in digital media (e.g., notes in news magazines), as well as through dissemination on social networks” [49]. The participants were recruited through social networks. The main social media used was Facebook, where a page called Salud Mental COVID (Mental Health COVID) was created for the project. On this Facebook page, there were shared advertisements for the study. Due to the relevance of the project, it had the support of the news media to be distributed to the general public during the initial phase of the pandemic. Some examples of the interviews conducted can be found in [50, 51].

4a-iii) Information giving during recruitment

Information given during recruitment. Specify how participants were briefed for recruitment and in the informed consent procedures (e.g., publish the informed consent documentation as appendix, see also item X26), as this information may have an effect on user self-selection, user expectation and may also bias results.

	1	2	3	4	5	
subitem not at all important	○	○	◉	○	○	essential

Clear selection

Does your paper address subitem 4a-iii?

Copy and paste relevant sections from the manuscript (include quotes in quotation marks "like this" to indicate direct quotes from your manuscript), or elaborate on this item by providing additional information not in the ms, or briefly explain why the item is not applicable/relevant for your study

Your answer

4b) Settings and locations where the data were collected

Your response is too large. Try shortening some answers.

Does your paper address CONSORT subitem 4b? *

Copy and paste relevant sections from the manuscript (include quotes in quotation marks "like this" to indicate direct quotes from your manuscript), or elaborate on this item by providing additional information not in the ms, or briefly explain why the item is not applicable/relevant for your study

The intervention was delivered to the Mexican population. However, as it was an open online intervention, participants from other countries could also access the platform.

4b-i) Report if outcomes were (self-)assessed through online questionnaires

Clearly report if outcomes were (self-)assessed through online questionnaires (as common in web-based trials) or otherwise.

	1	2	3	4	5	
subitem not at all important	○	○	◉	○	○	essential

Clear selection

Your response is too large. Try shortening some answers.

Does your paper address subitem 4b-i? *

Copy and paste relevant sections from the manuscript (include quotes in quotation marks "like this" to indicate direct quotes from your manuscript), or elaborate on this item by providing additional information not in the ms, or briefly explain why the item is not applicable/relevant for your study

Beck Depression Inventory second version (BDI-II) [55]. This instrument is a widely used 21-item self-report inventory measuring the severity of depression in adolescents and adults consistent with DSM IV criteria for depression.

The Generalized Anxiety Disorder 7-Item scale (GAD-7) [58].

The Scale of Post-Traumatic Stress Disorder (PTSD) traits is a self-report scale developed in Colombia by [60], which comprises five domains related to PTSD symptomatology in correspondence with DSM-V diagnosis criteria.

Widespread Fear Scale [62] Adapted by Chávez-Valdez [63].
This screening tool measures an emotional widespread fear such as fear of adversity in a particular context.

State-Trait Anxiety Inventory (STAI) (Spanish version [64]).
This instrument is a commonly used measure of trait and state anxiety. This inventory aims to distinguish between two types of anxiety. It can be used in clinical settings to diagnose anxiety and to distinguish it from depressive syndromes. This self-report measure categorizes symptoms related to anxiety as a personality trait (trait-anxiety) and distinguishes it from state anxiety, that is, transitory anxiety that a person experiences at an anxious specific unforeseen event (state-anxiety).

The Pittsburgh Sleep Quality Index (PSQI; [65]).
This self-administered questionnaire is a 19-item measure that assesses sleep quality and disturbances during the past month.

The Urban Insecurity Scale [67].
The Urban Insecurity Scale, is a self-report measure, the Spanish version named “Un nuevo instrumento de evaluación psicológica: el Cuestionario de Inseguridad Urbana (CIU)”was originally developed by [68].

The Scale for Suicide Ideation [70].
This scale aims to assess the frequency of attitudes, behaviors and plans to attempt suicide. It is divided into 19 items with response options of 0-2, giving a total score of 0-38, where a score ≥10 indicates an existing suicidal risk.

Your response is too large. Try shortening some answers.

4b-ii) Report how institutional affiliations are displayed

Report how institutional affiliations are displayed to potential participants [on ehealth media], as affiliations with prestigious hospitals or universities may affect volunteer rates, use, and reactions with regards to an intervention.(Not a required item – describe only if this may bias results)

	1	2	3	4	5	
subitem not at all important	○	◉	○	○	○	essential

Clear selection

Does your paper address subitem 4b-ii?

Copy and paste relevant sections from the manuscript (include quotes in quotation marks "like this" to indicate direct quotes from your manuscript), or elaborate on this item by providing additional information not in the ms, or briefly explain why the item is not applicable/relevant for your study

Your answer

5) The interventions for each group with sufficient details to allow replication, including how and when they were actually administered

5-i) Mention names, credential, affiliations of the developers, sponsors, and owners

Mention names, credential, affiliations of the developers, sponsors, and owners [6] (if authors/evaluators are owners or developer of the software, this needs to be declared in a “Conflict of interest” section or mentioned elsewhere in the manuscript).

	1	2	3	4	5	
subitem not at all important	○	○	○	◉	○	essential

Clear selection

Your response is too large. Try shortening some answers.

Does your paper address subitem 5-i?

Copy and paste relevant sections from the manuscript (include quotes in quotation marks "like this" to indicate direct quotes from your manuscript), or elaborate on this item by providing additional information not in the ms, or briefly explain why the item is not applicable/relevant for your study

2.8. Intervention
The intervention Mental Health COVID-19 (ITLAB)

5-ii) Describe the history/development process

Describe the history/development process of the application and previous formative evaluations (e.g., focus groups, usability testing), as these will have an impact on adoption/use rates and help with interpreting results.

	1	2	3	4	5	
subitem not at all important	○	○	◉	○	○	essential

Clear selection

Does your paper address subitem 5-ii?

Copy and paste relevant sections from the manuscript (include quotes in quotation marks "like this" to indicate direct quotes from your manuscript), or elaborate on this item by providing additional information not in the ms, or briefly explain why the item is not applicable/relevant for your study

Your answer

Your response is too large. Try shortening some answers.

5-iii) Revisions and updating

Revisions and updating. Clearly mention the date and/or version number of the application/intervention (and comparator, if applicable) evaluated, or describe whether the intervention underwent major changes during the evaluation process, or whether the development and/or content was “frozen” during the trial. Describe dynamic components such as news feeds or changing content which may have an impact on the replicability of the intervention (for unexpected events see item 3b).

	1	2	3	4	5	
subitem not at all important	○	○	◉	○	○	essential

Clear selection

Does your paper address subitem 5-iii?

Copy and paste relevant sections from the manuscript (include quotes in quotation marks "like this" to indicate direct quotes from your manuscript), or elaborate on this item by providing additional information not in the ms, or briefly explain why the item is not applicable/relevant for your study

Your answer

5-iv) Quality assurance methods

Provide information on quality assurance methods to ensure accuracy and quality of information provided [1], if applicable.

	1	2	3	4	5	
subitem not at all important	○	○	◉	○	○	essential

Clear selection

Your response is too large. Try shortening some answers.

Does your paper address subitem 5-iv?

Copy and paste relevant sections from the manuscript (include quotes in quotation marks "like this" to indicate direct quotes from your manuscript), or elaborate on this item by providing additional information not in the ms, or briefly explain why the item is not applicable/relevant for your study

Your answer

5-v) Ensure replicability by publishing the source code, and/or providing screenshots/screen-capture video, and/or providing flowcharts of the algorithms used

Ensure replicability by publishing the source code, and/or providing screenshots/screen-capture video, and/or providing flowcharts of the algorithms used. Replicability (i.e., other researchers should in principle be able to replicate the study) is a hallmark of scientific reporting.

	1	2	3	4	5	
subitem not at all important	○	○	◉	○	○	essential

Clear selection

Does your paper address subitem 5-v?

Copy and paste relevant sections from the manuscript (include quotes in quotation marks "like this" to indicate direct quotes from your manuscript), or elaborate on this item by providing additional information not in the ms, or briefly explain why the item is not applicable/relevant for your study

For a detailed intervention description, please read the full protocol [49].

Data availability statement
The database is available by reasonable request by contacting the corresponding author.

Your response is too large. Try shortening some answers.

5-vi) Digital preservation

Digital preservation: Provide the URL of the application, but as the intervention is likely to change or disappear over the course of the years; also make sure the intervention is archived (Internet Archive, webcitation.org, and/or publishing the source code or screenshots/videos alongside the article). As pages behind login screens cannot be archived, consider creating demo pages which are accessible without login.

	1	2	3	4	5	
subitem not at all important	○	○	○	◉	○	essential

Clear selection

Does your paper address subitem 5-vi?

Copy and paste relevant sections from the manuscript (include quotes in quotation marks "like this" to indicate direct quotes from your manuscript), or elaborate on this item by providing additional information not in the ms, or briefly explain why the item is not applicable/relevant for your study

Footnotes
1 www.saludmentalcovid.com

5-vii) Access

Access: Describe how participants accessed the application, in what setting/context, if they had to pay (or were paid) or not, whether they had to be a member of specific group. If known, describe how participants obtained “access to the platform and Internet” [1]. To ensure access for editors/reviewers/readers, consider to provide a “backdoor” login account or demo mode for reviewers/readers to explore the application (also important for archiving purposes, see vi).

	1	2	3	4	5	
subitem not at all important	○	○	◉	○	○	essential

Clear selection

Your response is too large. Try shortening some answers.

Does your paper address subitem 5-vii? *

Copy and paste relevant sections from the manuscript (include quotes in quotation marks "like this" to indicate direct quotes from your manuscript), or elaborate on this item by providing additional information not in the ms, or briefly explain why the item is not applicable/relevant for your study

The intervention was delivered to the Mexican population. However, as it was an open online intervention, participants from other countries could also access the platform.

5-viii) Mode of delivery, features/functionalities/components of the intervention and comparator, and the theoretical framework

Describe mode of delivery, features/functionalities/components of the intervention and comparator, and the theoretical framework [6] used to design them (instructional strategy [1], behaviour change techniques, persuasive features, etc., see e.g., [7, 8] for terminology). This includes an in-depth description of the content (including where it is coming from and who developed it) [1],” whether [and how] it is tailored to individual circumstances and allows users to track their progress and receive feedback” [6]. This also includes a description of communication delivery channels and – if computer-mediated communication is a component – whether communication was synchronous or asynchronous [6]. It also includes information on presentation strategies [1], including page design principles, average amount of text on pages, presence of hyperlinks to other resources, etc. [1].

	1	2	3	4	5	
subitem not at all important	○	○	○	◉	○	essential

Clear selection

Your response is too large. Try shortening some answers.

Does your paper address subitem 5-viii? *

Copy and paste relevant sections from the manuscript (include quotes in quotation marks "like this" to indicate direct quotes from your manuscript), or elaborate on this item by providing additional information not in the ms, or briefly explain why the item is not applicable/relevant for your study

The intervention Mental Health COVID-19 (ITLAB) comprises 15 modules, of which 11 are centered on positive psychology (e.g., to provide tools to recognize personal abilities to recover after a stressful event), two on CBT (e.g., the importance of emotions, and why they are experienced), and two on BAT (e.g., performing a physical exercise that involves motor skills of the body). The intervention was delivered mainly in two formats:1) videos that were uploaded on YouTube and embedded on the platform, and 2) PDF files that the participant could download that included further information about the session along with exercises and examples. The content of each video is presented in Table 1. At the end of each module, the platform presented the participant with a five-question survey with multiple-choice answers that evaluated the knowledge acquired in the module. The intervention was self-paced, meaning the participants could conclude the modules according to their time disposition. For a detailed intervention description, please read the full protocol [49].

Participants were randomly assigned to one of two groups:
1) Control Active Comparator: Self-guided online intervention (SGOI) without assistance via chat, and
2) Experimental: Self-guided online intervention plus chat support (SGOI+C) provided by therapists in training and supervised by two authors of this study. The chat support was open for the users on the SGOI+C group to contact for any questions regarding the platform and the intervention.

5-ix) Describe use parameters

Describe use parameters (e.g., intended “doses” and optimal timing for use). Clarify what instructions or recommendations were given to the user, e.g., regarding timing, frequency, heaviness of use, if any, or was the intervention used ad libitum.

	1	2	3	4	5	
subitem not at all important	○	○	◉	○	○	essential

Clear selection

Your response is too large. Try shortening some answers.

Does your paper address subitem 5-ix?

Copy and paste relevant sections from the manuscript (include quotes in quotation marks "like this" to indicate direct quotes from your manuscript), or elaborate on this item by providing additional information not in the ms, or briefly explain why the item is not applicable/relevant for your study

For a detailed intervention description, please read the full protocol [49].

5-x) Clarify the level of human involvement

Clarify the level of human involvement (care providers or health professionals, also technical assistance) in the e-intervention or as co-intervention (detail number and expertise of professionals involved, if any, as well as "type of assistance offered, the timing and frequency of the support, how it is initiated, and the medium by which the assistance is delivered". It may be necessary to distinguish between the level of human involvement required for the trial, and the level of human involvement required for a routine application outside of a RCT setting (discuss under item 21 – generalizability).

	1	2	3	4	5	
subitem not at all important	○	○	○	◉	○	essential

Clear selection

Does your paper address subitem 5-x?

Copy and paste relevant sections from the manuscript (include quotes in quotation marks "like this" to indicate direct quotes from your manuscript), or elaborate on this item by providing additional information not in the ms, or briefly explain why the item is not applicable/relevant for your study

As described in Arenas-Landgrave et al, [48], a personalized attention chat was available on the platform 'Salud Mental COVID', where the user could communicate with a mental health specialist upon entering the system. This staff was supervised by AR-G and PAL. AD-R provided training to the therapists on using the chat service and monitored the proper use of this tool. The needs addressed in the chat could be emotional support, which refers to those occasions in which the person described symptoms of uncontrollable emotional discomfort; technical guidance about the platform or any of the modules; or referral to other sources of assistance in case of requiring more specialized care.

Your response is too large. Try shortening some answers.

5-xi) Report any prompts/reminders used

Report any prompts/reminders used: Clarify if there were prompts (letters, emails, phone calls, SMS) to use the application, what triggered them, frequency etc. It may be necessary to distinguish between the level of prompts/reminders required for the trial, and the level of prompts/reminders for a routine application outside of a RCT setting (discuss under item 21 – generalizability).

	1	2	3	4	5	
subitem not at all important	○	○	◉	○	○	essential

Clear selection

Does your paper address subitem 5-xi? *

Copy and paste relevant sections from the manuscript (include quotes in quotation marks "like this" to indicate direct quotes from your manuscript), or elaborate on this item by providing additional information not in the ms, or briefly explain why the item is not applicable/relevant for your study

The intervention did not specify this

5-xii) Describe any co-interventions (incl. training/support)

Describe any co-interventions (incl. training/support): Clearly state any interventions that are provided in addition to the targeted eHealth intervention, as ehealth intervention may not be designed as stand-alone intervention. This includes training sessions and support [1]. It may be necessary to distinguish between the level of training required for the trial, and the level of training for a routine application outside of a RCT setting (discuss under item 21 – generalizability.

	1	2	3	4	5	
subitem not at all important	○	○	◉	○	○	essential

Clear selection

Your response is too large. Try shortening some answers.

Does your paper address subitem 5-xii? *

Copy and paste relevant sections from the manuscript (include quotes in quotation marks "like this" to indicate direct quotes from your manuscript), or elaborate on this item by providing additional information not in the ms, or briefly explain why the item is not applicable/relevant for your study

There was no co-intervention included

6a) Completely defined pre-specified primary and secondary outcome measures, including how and when they were assessed

Your response is too large. Try shortening some answers.

Does your paper address CONSORT subitem 6a? *

Copy and paste relevant sections from the manuscript (include quotes in quotation marks "like this" to indicate direct quotes from your manuscript), or elaborate on this item by providing additional information not in the ms, or briefly explain why the item is not applicable/relevant for your study

Your response is too large. Try shortening some answers.

2.7. Instruments
2.7.1. Primary Outcome Measures
Beck Depression Inventory second version (BDI-II) [55].
This instrument is a widely used 21-item self-report inventory measuring the severity of depression in adolescents and adults consistent with DSM IV criteria for depression. The response options range from 0 to 3, except for items 16 and 18, which have seven response options each. Total scores range from 0-63, where 0-13 points indicate minimal depression, 14-19 indicates mild depression, 20-28 indicates moderate depression, and 26-63 indicates severe depression. Studies of the psychometric properties of the Spanish version of the BDI-II for the Mexican population were conducted by [56] and [57] for version II, showing adequate concurrent validity (r≥ .66) and reliability (Cronbach alpha values between .87 and .92) coefficients.

The Generalized Anxiety Disorder 7-Item scale (GAD-7) [58].
This instrument is a screening tool for generalized anxiety. The GAD-7 is a brief scale that consists of 7 items designed to measure the severity of symptoms of Generalized Anxiety Disorder (GAD). When screening for anxiety disorders, a score of 8 or greater represents a reasonable cut-off point for identifying possible cases of GAD. Using a cut-off of 8, the GAD-7 reached good sensitivity and specificity. The maximum total score is 21. A score between 0 and 4 indicates that anxiety is not perceived, and a score between 15 and 21 shows perceived severe anxiety. Some items assess feeling nervous or anxious, inability to stop or control worrying, restlessness, and being easily annoyed. The questions in this scale are answered with scores ranging from 0 (never) to 3 (nearly every day). The version by [59] was used for this study, which shows adequate concurrent validity (r≥ 0.70) and reliability (Cronbach alpha 0.93) coefficients.

Scale of Post-traumatic Stress Traits in the Mexican Youth Exposed to Social Violence [60].
The Scale of Post-Traumatic Stress Disorder (PTSD) traits is a self-report scale developed in Colombia by [60], which comprises five domains related to PTSD symptomatology in correspondence with DSM-V diagnosis criteria. The symptoms include having negative alterations in cognition and mood (criterion D), intrusion symptoms (criterion B), functional significance (criterion G), avoidance (criterion C), and arousal and reactivity alterations (criterion E). The scale rates the presence/absence of the above mentioned discrete categories. Chávez-Valdez et al. [61] validated the scale to diagnose PTSD symptomatology based on DSM-IV criteria in Mexico. The scale consists of 24 items (e.g., “Most of the time, I avoid the things and places that remind me of the situation”), with scores ranging from 1 (totally disagree) to 4 (totally agree). This is a discrete-categorical scale, with item loadings that range between .37 to .87. Correlations between factors ranged from r = .45 to r = .85, and good internal consistency of the Cronbach´s alpha between .92 and .97 [61] was found.

Widespread Fear Scale [62] Adapted by Chávez-Valdez [63].
This screening tool measures an emotional widespread fear such as fear of adversity in a particular context. It is composed of seven items, with options of 0 = nothing to 3 = a lot.

In previous studies done by Ruiz-Pérez [62] in Colombia, an acceptable internal consistency of 0.90 was reached. It consists of several items about the fear of being a victim of the context and three items about being afraid of the neighborhood neighbourhood [illegible]

Your response is too large. Try shortening some answers.

In this way, there is convergent validity between the two scales. In turn, the divergent validity of the Social Insecurity Perception Scale was carried out because it contains a factor that

measures the perception of citizen uncertainty through the widespread fear, which measures, in the opinion of the interviewer, the possibility of a perception of private uncertainties, which measures the opposite construct with an effect size (r = -.28, p =.001). The adaptation of the instrument, performed in northern Mexico by Chávez-Valdez [63] , obtained a Cronbach alpha coefficient of .92.

State-Trait Anxiety Inventory (STAI) (Spanish version [64]).
This instrument is a commonly used measure of trait and state anxiety. This inventory aims to distinguish between two types of anxiety. It can be used in clinical settings to diagnose anxiety and to distinguish it from depressive syndromes. This self-report measure categorizes symptoms related to anxiety as a personality trait (trait-anxiety) and distinguishes it from state anxiety, that is, transitory anxiety that a person experiences at an anxious specific unforeseen event (state-anxiety). It is composed of 40 items, 20 for the state and 20 for the trait. Internal consistency ranged from .86 to .95 [64].

The Pittsburgh Sleep Quality Index (PSQI; [65]).
This self-administered questionnaire is a 19-item measure that assesses sleep quality and disturbances during the past month. These items are rated considering the frequency or severity of sleep disturbance using scores ranging from 0 (not during the past month) to 3 (three or more times a week). Items combine to form 7 components: sleep duration, sleep disturbance, sleep latency, daytime dysfunction, sleep efficiency, overall quality of sleep, and use of sleep medication [65].The total score of the instrument is obtained by the sum of the component scores ranging from 0 to 21, with higher scores representing lower sleep quality. The evaluation of the Mexican population showed solid validity (r≥ 0.53) and reliability (α=.78) coefficients [66].

The Urban Insecurity Scale [67].
The Urban Insecurity Scale, is a self-report measure, the Spanish version named "Un nuevo instrumento de evaluación psicológica: el Cuestionario de Inseguridad Urbana (CIU)"was originally developed by [68]. This instrument has been designed based on a version of the Inventory of Anxiety Situations and Responses (IASR), a Spanish scale named Inventario de Situaciones y Respuestas de Ansiedad (ISRA) proposed by Miguel-Tobal and Cano-Vindel in its first version since 1997 [69]. This scale comprises 15 items grouped into four dimensions characterized by reactions that indicate certain affective elements, such as worry, fear, feelings of insecurity, physiological activation, cognitive confrontation, and behavioral promotion components. The assessment of the test is made by adding the scores given by the person to each item of each scale. Thus, four scores are obtained: Affective, Cognitive, Physiological, and Behavioral, which represent the scores of each of the systems of answer. The total score is obtained by adding the four components previously described. For the interpretation of the profile, four levels of stress (or anxiety) have been defined: 1) Absence of stress or normal stress, 2) Moderate stress, 3) Severe stress, and 4) Extreme or post-traumatic stress. In the reliability analysis, the scale reached Cronbach's alpha of 0.92 [67]. In this study, the alpha coefficient reached 0.91.

The Scale for Suicide Ideation [70].
This scale aims to assess the frequency of attitudes, behaviors and plans to attempt suicide. It is divided into 19 items with response options of 0-2, giving a total score of 0-38, where a score ≥10 indicates an existing suicidal risk. This scale has been validated by [71] in the Mexican population, obtaining a Cronbach alpha of .84. For this study, only two items

Your response is too large. Try shortening some answers.

2.7.2. Secondary Outcome Measures
2.7.2.1. Acceptance, Satisfaction, and Usability Measures

Opinion about the treatment [72].
This questionnaire comprises four questions that report the participants' level of satisfaction with the treatment. The participants can report if they would recommend the treatment to a friend or family member, if they consider it helpful, and if they think that the treatment was difficult to manage or aversive. The questions are answered on a scale from 1 (nothing) to 10 (very much).

System Usability Scale [73].
The System Usability Scale (SUS) is an instrument that is designed to validate the usability of a system. It comprises 10 items, which are answered on a 5-point Likert-type scale concerning the degree of conformity of the product (1 completely disagree to 5 completely agree). All values must be added together and multiplied by 2.5 to obtain this scale's global score, which ranges between 0 and 100. The SUS is a widely used standardized questionnaire, translated into many languages, such as Arabic, and Polish, among others [74]. Some items are: "I think that I would like to use this system frequently", and, "I needed to learn a lot of things before I could get going with this system".

6a-i) Online questionnaires: describe if they were validated for online use and apply CHERRIES items to describe how the questionnaires were designed/deployed

If outcomes were obtained through online questionnaires, describe if they were validated for online use and apply CHERRIES items to describe how the questionnaires were designed/deployed [9].

	1	2	3	4	5	
subitem not at all important	○	○	◉	○	○	essential

Clear selection

Does your paper address subitem 6a-i?

Copy and paste relevant sections from manuscript text

Your answer

Your response is too large. Try shortening some answers.

6a-ii) Describe whether and how “use” (including intensity of use/dosage) was defined/measured/monitored

Describe whether and how “use” (including intensity of use/dosage) was defined/measured/monitored (logins, logfile analysis, etc.). Use/adoption metrics are important process outcomes that should be reported in any ehealth trial.

	1	2	3	4	5	
subitem not at all important	○	○	◉	○	○	essential

Clear selection

Does your paper address subitem 6a-ii?

Copy and paste relevant sections from manuscript text

Your answer

6a-iii) Describe whether, how, and when qualitative feedback from participants was obtained

Describe whether, how, and when qualitative feedback from participants was obtained (e.g., through emails, feedback forms, interviews, focus groups).

	1	2	3	4	5	
subitem not at all important	○	○	○	◉	○	essential

Clear selection

Does your paper address subitem 6a-iii?

Copy and paste relevant sections from manuscript text

Your answer

Your response is too large. Try shortening some answers.

Does your paper address CONSORT subitem 6b? *

Copy and paste relevant sections from the manuscript (include quotes in quotation marks "like this" to indicate direct quotes from your manuscript), or elaborate on this item by providing additional information not in the ms, or briefly explain why the item is not applicable/relevant for your study

The study has some limitations. The main limitation is that due to the small sample size for the SAOI group, the planned analysis was modified, which corresponded to the evaluation of the second hypothesis on the moderating role of coping strategies, acceptance, and satisfaction variables on clinical change by group. Initially, it was intended to be done separately for each group (SAOI and SAOI+C), but instead, it was performed considering the total sample. Furthermore, it was not possible to assess whether the changes were maintained for the SAOI group, as none completed follow-up at 3 or 6 months.

Finally, psychotic disorder was proposed as an exclusion criterion in the study protocol. Nevertheless, we did not use this criterion when we conducted the study. We mention here this inconsistency as a limitation of this research. Further interventions aimed at the general population should explore the presence of psychotic symptoms.

7a) How sample size was determined

NPT: When applicable, details of whether and how the clustering by care provides or centers was addressed

7a-i) Describe whether and how expected attrition was taken into account when calculating the sample size

Describe whether and how expected attrition was taken into account when calculating the sample size.

	1	2	3	4	5	
subitem not at all important	○	○	○	●	○	essential

Clear selection

Your response is too large. Try shortening some answers.

Does your paper address subitem 7a-i?

Copy and paste relevant sections from manuscript title (include quotes in quotation marks "like this" to indicate direct quotes from your manuscript), or elaborate on this item by providing additional information not in the ms, or briefly explain why the item is not applicable/relevant for your study

2.5. Sample Size
The calculated sample size for this study was 166 participants (83 per group). Further study details are available in the protocol manuscript by Dominguez-Rodriguez et al., [49]. The sample size was considered based on the effect sizes in controlled clinical studies in which the efficacy of web-based psychological interventions was evaluated. For the present study, the Cohen d index was used, assuming that the variances of the two groups were homogeneous.
Furthermore, the study included two conditions: an a priori analysis to compare the means between the two independent groups and a conservative approach to include an effect size with an average magnitude of 0.25 (Cohen d, equivalent to g=0.5), a significance level (α) of .05 (P<.05, which corresponds to 95% confidence), and a conventional statistical power of 80% (1 – β = 0.8). For the analysis, the software G*Power version 3.1.6 was used, and a required sample size of 128 participants was obtained (64 per group).
However, the number of participants was increased by 30% to control the variable related to dropping out of participants during the treatment; this rate is reported in the literature on web-based treatments [53, 54]. Thus, the total required sample size will be 166 participants (83 per group).

7b) When applicable, explanation of any interim analyses and stopping guidelines

Does your paper address CONSORT subitem 7b? *

Copy and paste relevant sections from the manuscript (include quotes in quotation marks "like this" to indicate direct quotes from your manuscript), or elaborate on this item by providing additional information not in the ms, or briefly explain why the item is not applicable/relevant for your study

The study did not include any interim analysis or stopping guidelines

8a) Method used to generate the random allocation sequence

NPT: When applicable, how care providers were allocated to each trial group

Your response is too large. Try shortening some answers.

Does your paper address CONSORT subitem 8a? *

Copy and paste relevant sections from the manuscript (include quotes in quotation marks "like this" to indicate direct quotes from your manuscript), or elaborate on this item by providing additional information not in the ms, or briefly explain why the item is not applicable/relevant for your study

2.6. Randomization
Once the evaluation was completed, the users were randomly assigned to one of the study conditions. The randomization was performed by an independent researcher using web-based randomization algorithm at a ratio of 1:1 using the method of randomly permuted blocks.

8b) Type of randomisation; details of any restriction (such as blocking and block size)

Does your paper address CONSORT subitem 8b? *

Copy and paste relevant sections from the manuscript (include quotes in quotation marks "like this" to indicate direct quotes from your manuscript), or elaborate on this item by providing additional information not in the ms, or briefly explain why the item is not applicable/relevant for your study

The randomization was performed by an independent researcher using web-based randomization algorithm at a ratio of 1:1 using the method of randomly permuted blocks.

9) Mechanism used to implement the random allocation sequence (such as sequentially numbered containers), describing any steps taken to conceal the sequence until interventions were assigned

Your response is too large. Try shortening some answers.

Does your paper address CONSORT subitem 9? *

Copy and paste relevant sections from the manuscript (include quotes in quotation marks "like this" to indicate direct quotes from your manuscript), or elaborate on this item by providing additional information not in the ms, or briefly explain why the item is not applicable/relevant for your study

using web-based randomization algorithm at a ratio of 1:1

10) Who generated the random allocation sequence, who enrolled participants, and who assigned participants to interventions

Does your paper address CONSORT subitem 10? *

Copy and paste relevant sections from the manuscript (include quotes in quotation marks "like this" to indicate direct quotes from your manuscript), or elaborate on this item by providing additional information not in the ms, or briefly explain why the item is not applicable/relevant for your study

an independent researcher

11a) If done, who was blinded after assignment to interventions (for example, participants, care providers, those assessing outcomes) and how

NPT: Whether or not administering co-interventions were blinded to group assignment

11a-i) Specify who was blinded, and who wasn't

Specify who was blinded, and who wasn't. Usually, in web-based trials it is not possible to blind the participants [1, 3] (this should be clearly acknowledged), but it may be possible to blind outcome assessors, those doing data analysis or those administering co-interventions (if any).

	1	2	3	4	5	
subitem not at all important	○	○	○	●	○	essential

Clear selection

Your response is too large. Try shortening some answers.

Does your paper address subitem 11a-i? *

Copy and paste relevant sections from the manuscript (include quotes in quotation marks "like this" to indicate direct quotes from your manuscript), or elaborate on this item by providing additional information not in the ms, or briefly explain why the item is not applicable/relevant for your study

The participants were unaware that there was an intervention group and a comparison group, and they were unrelated.

11a-ii) Discuss e.g., whether participants knew which intervention was the “intervention of interest” and which one was the “comparator”

Informed consent procedures (4a-ii) can create biases and certain expectations - discuss e.g., whether participants knew which intervention was the “intervention of interest” and which one was the “comparator”.

	1	2	3	4	5	
subitem not at all important	○	○	○	◉	○	essential

Clear selection

Does your paper address subitem 11a-ii?

Copy and paste relevant sections from the manuscript (include quotes in quotation marks "like this" to indicate direct quotes from your manuscript), or elaborate on this item by providing additional information not in the ms, or briefly explain why the item is not applicable/relevant for your study

The participants were unaware that there was an intervention group and a comparison group, and they were unrelated.

11b) If relevant, description of the similarity of interventions

(this item is usually not relevant for ehealth trials as it refers to similarity of a placebo or sham intervention to a active medication/intervention)

Your response is too large. Try shortening some answers.

Does your paper address CONSORT subitem 11b? *

Copy and paste relevant sections from the manuscript (include quotes in quotation marks "like this" to indicate direct quotes from your manuscript), or elaborate on this item by providing additional information not in the ms, or briefly explain why the item is not applicable/relevant for your study

1) Control Active Comparator: Self-guided online intervention (SGOI) without assistance via chat, and
2) Experimental: Self-guided online intervention plus chat support (SGOI+C) provided by therapists in training and supervised by two authors of this study. The chat support was open for the users on the SGOI+C group to contact for any questions regarding the platform and the intervention.

12a) Statistical methods used to compare groups for primary and secondary outcomes

NPT: When applicable, details of whether and how the clustering by care providers or centers was addressed

Your response is too large. Try shortening some answers.

Does your paper address CONSORT subitem 12a? *

Copy and paste relevant sections from the manuscript (include quotes in quotation marks "like this" to indicate direct quotes from your manuscript), or elaborate on this item by providing additional information not in the ms, or briefly explain why the item is not applicable/relevant for your study

2.9. Data analysis
Descriptive analysis of sample sociodemographic characteristics and clinical parameters at baseline were reported through the median and interquartile range for continuous variables to test for differences between two independent groups using non-parametric techniques, such as the Mann-Whitney U Test. Frequency and percentages were reported for categorical variables. Differences between intervention groups were assessed through Mann-Whitney's U for continuous variables and the Chi-Squared test for categorical variables. Changes due to intervention between the pretest and posttest and 3- and 6-month follow-up assessments were analyzed with Wilcoxon's W test. They were applied to the SGOI+C group since the SGOI group did not answer the follow-up assessment invitations. As the post-intervention assessment was only available to those who completed the entire treatment, all participants who had only accessed part of the modules were excluded from these analyses. Cases with missing values were excluded from each statistical test, so a maximum of n=31 was obtained for the pre-and post-analyses, n=18 in the 3-month follow-up, and n=4 in the 6-month follow-up. The size effect for the statistically significant differences obtained from the comparative analyses was estimated using Rosenthal's R [75].

12a-i) Imputation techniques to deal with attrition / missing values

Imputation techniques to deal with attrition / missing values: Not all participants will use the intervention/comparator as intended and attrition is typically high in ehealth trials. Specify how participants who did not use the application or dropped out from the trial were treated in the statistical analysis (a complete case analysis is strongly discouraged, and simple imputation techniques such as LOCF may also be problematic [4]).

	1	2	3	4	5	
subitem not at all important	○	○	○	◉	○	essential

Clear selection

Your response is too large. Try shortening some answers.

Does your paper address subitem 12a-i? *

Copy and paste relevant sections from the manuscript (include quotes in quotation marks "like this" to indicate direct quotes from your manuscript), or elaborate on this item by providing additional information not in the ms, or briefly explain why the item is not applicable/relevant for your study

Cases with missing values were excluded from each statistical test, so a maximum of n=31 was obtained for the pre-and post-analyses, n=18 in the 3-month follow-up, and n=4 in the 6-month follow-up. The size effect for the statistically significant differences obtained from the comparative analyses was estimated using Rosenthal's R [75].

12b) Methods for additional analyses, such as subgroup analyses and adjusted analyses

Does your paper address CONSORT subitem 12b? *

Copy and paste relevant sections from the manuscript (include quotes in quotation marks "like this" to indicate direct quotes from your manuscript), or elaborate on this item by providing additional information not in the ms, or briefly explain why the item is not applicable/relevant for your study

Moderating regression analysis was performed to test the hypothesized moderating role of coping strategies, usability, and opinion about treatment on clinical change. Only those variables showing statistically significant change were introduced as independent and dependent variables. Pretest scores of clinical variables were introduced as independent variables, posttest scores as dependent variables, and total scores of The Urban Insecurity Scale, System Usability Scale, and Opinion Questionnaire about Treatment were introduced as moderating variables. In addition, sociodemographic variables (age, gender, and educational attainment) and the assignment to the SGOI or SGOI+C group were added to the model as control variables.

The model was built from 50,000 bootstrapping samples. The Johnson-Neyman interval was computed in order to identify the points on the slope at which there are significant changes in the effect of the moderator. All analyses were carried out using Statistical Package for Social Sciences (SPSS) v. 26. For moderating analysis, the macro PROCESS was used (www.processmacro.org).

[illegible]

Your response is too large. Try shortening some answers.

X26-i) Comment on ethics committee approval

	1	2	3	4	5	
subitem not at all important	○	○	○	○	◉	essential

Clear selection

Does your paper address subitem X26-i?

Copy and paste relevant sections from the manuscript (include quotes in quotation marks "like this" to indicate direct quotes from your manuscript), or elaborate on this item by providing additional information not in the ms, or briefly explain why the item is not applicable/relevant for your study

Ethical Approval
This study was approved by the ethics committee of the Escuela Libre de Psicología, Universidad de Ciencias del Comportamiento (Ethics Committee of the Free School of Psychology University of Behavioral Sciences) in Chihuahua, Mexico (reference number Folio 2008), and registered in Clinical Trials: NCT04468893, and in the International Registered Report Identifier (IRRID): DERR1-10.2196/23117.

x26-ii) Outline informed consent procedures

Outline informed consent procedures e.g., if consent was obtained offline or online (how? Checkbox, etc.?), and what information was provided (see 4a-ii). See [6] for some items to be included in informed consent documents.

	1	2	3	4	5	
subitem not at all important	○	○	◉	○	○	essential

Clear selection

Your response is too large. Try shortening some answers.

Does your paper address subitem X26-ii?

Copy and paste relevant sections from the manuscript (include quotes in quotation marks "like this" to indicate direct quotes from your manuscript), or elaborate on this item by providing additional information not in the ms, or briefly explain why the item is not applicable/relevant for your study

Your answer

X26-iii) Safety and security procedures

Safety and security procedures, incl. privacy considerations, and any steps taken to reduce the likelihood or detection of harm (e.g., education and training, availability of a hotline)

	1	2	3	4	5	
subitem not at all important	○	○	◉	○	○	essential

Clear selection

Does your paper address subitem X26-iii?

Copy and paste relevant sections from the manuscript (include quotes in quotation marks "like this" to indicate direct quotes from your manuscript), or elaborate on this item by providing additional information not in the ms, or briefly explain why the item is not applicable/relevant for your study

Your answer

RESULTS

13a) For each group, the numbers of participants who were randomly assigned, received intended treatment, and were analysed for the primary outcome

NPT: The number of care providers or centers performing the intervention in each group and the number of patients treated by each care provider in each center

Your response is too large. Try shortening some answers.

Does your paper address CONSORT subitem 13a? *

Copy and paste relevant sections from the manuscript (include quotes in quotation marks "like this" to indicate direct quotes from your manuscript), or elaborate on this item by providing additional information not in the ms, or briefly explain why the item is not applicable/relevant for your study

In total, 2,047 participants underwent the eligibility assessment. From which, 1,439 were excluded. Exclusion reasons were the following: not email account confirmation (n=554), not accepting informed consent (n=346), incomplete initial assessment (n=499), and not from Mexico (n=40). From the remaining 608 randomized participants to one of the two groups (see Figure 1), 266 did not complete any module. Of the remaining participants, several dropped out during the intervention. , being aAt the start of the intervention, the main modules were where the participants dropped out. Table 2 presents this information in detail.

13b) For each group, losses and exclusions after randomisation, together with reasons

Does your paper address CONSORT subitem 13b? (NOTE: Preferably, this is shown in a CONSORT flow diagram) *

Copy and paste relevant sections from the manuscript (include quotes in quotation marks "like this" to indicate direct quotes from your manuscript), or elaborate on this item by providing additional information not in the ms, or briefly explain why the item is not applicable/relevant for your study

In total, 2,047 participants underwent the eligibility assessment. From which, 1,439 were excluded. Exclusion reasons were the following: not email account confirmation (n=554), not accepting informed consent (n=346), incomplete initial assessment (n=499), and not from Mexico (n=40). From the remaining 608 randomized participants to one of the two groups (see Figure 1), 266 did not complete any module. Of the remaining participants, several dropped out during the intervention. , being aAt the start of the intervention, the main modules were where the participants dropped out. Table 2 presents this information in detail.

Your response is too large. Try shortening some answers.

13b-i) Attrition diagram

Strongly recommended: An attrition diagram (e.g., proportion of participants still logging in or using the intervention/comparator in each group plotted over time, similar to a survival curve) or other figures or tables demonstrating usage/dose/engagement.

	1	2	3	4	5	
subitem not at all important	○	○	○	◉	○	essential

Clear selection

Does your paper address subitem 13b-i?

Copy and paste relevant sections from the manuscript or cite the figure number if applicable (include quotes in quotation marks "like this" to indicate direct quotes from your manuscript), or elaborate on this item by providing additional information not in the ms, or briefly explain why the item is not applicable/relevant for your study

From the remaining 608 randomized participants to one of the two groups (see Figure 1), 266 did not complete any module.

14a) Dates defining the periods of recruitment and follow-up

Does your paper address CONSORT subitem 14a? *

Copy and paste relevant sections from the manuscript (include quotes in quotation marks "like this" to indicate direct quotes from your manuscript), or elaborate on this item by providing additional information not in the ms, or briefly explain why the item is not applicable/relevant for your study

The intervention started in May 2020, and the data was collected until June 2022

Your response is too large. Try shortening some answers.

14a-i) Indicate if critical “secular events” fell into the study period

Indicate if critical “secular events” fell into the study period, e.g., significant changes in Internet resources available or “changes in computer hardware or Internet delivery resources”

	1	2	3	4	5	
subitem not at all important	○	○	◉	○	○	essential

Clear selection

Does your paper address subitem 14a-i?

Copy and paste relevant sections from the manuscript (include quotes in quotation marks "like this" to indicate direct quotes from your manuscript), or elaborate on this item by providing additional information not in the ms, or briefly explain why the item is not applicable/relevant for your study

The reason for the long period of data collection is due to the COVID-19 restrictions and health measures that were still effective in Mexico. Also, as in many developing countries, the vaccination was slower due to reduced vaccines available, and in the case of Mexico, the vaccination process was not equally distributed, with areas with higher vaccination percentages, such as Baja California Norte and Mexico City, compared to the rest of the country [52], and this could also have an impact on mental health

14b) Why the trial ended or was stopped (early)

Does your paper address CONSORT subitem 14b? *

Copy and paste relevant sections from the manuscript (include quotes in quotation marks "like this" to indicate direct quotes from your manuscript), or elaborate on this item by providing additional information not in the ms, or briefly explain why the item is not applicable/relevant for your study

There is no mention in the manuscript since the trial did not stoped earlier

Your response is too large. Try shortening some answers.

15) A table showing baseline demographic and clinical characteristics for each group

NPT: When applicable, a description of care providers (case volume, qualification, expertise, etc.) and centers (volume) in each group

Does your paper address CONSORT subitem 15? *

Copy and paste relevant sections from the manuscript (include quotes in quotation marks "like this" to indicate direct quotes from your manuscript), or elaborate on this item by providing additional information not in the ms, or briefly explain why the item is not applicable/relevant for your study

3.1 Sociodemographic characteristics of the sample
Fifty-two participants completed the intervention; however, 16 did not complete the post-assessment, so they were discarded from the analysis. Thirty-six participants completed the intervention, including the pre and post-assessment. Twenty-nine (80.6%) were female, and seven (19.4%) were male. More than half of the participants held a university degree (n=21; 58.3%), seven (19.4%) had a master's degree, six (16.7%) had high school studies, one had middle school studies (2.8%), and one (2.8%) declared to have other academic attainments. Two-thirds (n=24; 66.7%) were working at the beginning of the intervention. The median age was 35 years (IQR 37). For more details, see Table 3. Of the 36 participants who completed the intervention, 31 (86.1%) received the self-guided online intervention plus chat assistance (SGOI+C), whereas five participants (13.9%) received it without chat support (SGOI).

15-i) Report demographics associated with digital divide issues

In ehealth trials it is particularly important to report demographics associated with digital divide issues, such as age, education, gender, social-economic status, computer/Internet/ehealth literacy of the participants, if known.

	1	2	3	4	5	
subitem not at all important	○	○	●	○	○	essential

Clear selection

Your response is too large. Try shortening some answers.

Does your paper address subitem 15-i? *

Copy and paste relevant sections from the manuscript (include quotes in quotation marks "like this" to indicate direct quotes from your manuscript), or elaborate on this item by providing additional information not in the ms, or briefly explain why the item is not applicable/relevant for your study

3.1 Sociodemographic characteristics of the sample
Fifty-two participants completed the intervention; however, 16 did not complete the post-assessment, so they were discarded from the analysis. Thirty-six participants completed the intervention, including the pre and post-assessment. Twenty-nine (80.6%) were female, and seven (19.4%) were male. More than half of the participants held a university degree (n=21; 58.3%), seven (19.4%) had a master's degree, six (16.7%) had high school studies, one had middle school studies (2.8%), and one (2.8%) declared to have other academic attainments. Two-thirds (n=24; 66.7%) were working at the beginning of the intervention. The median age was 35 years (IQR 37). For more details, see Table 3. Of the 36 participants who completed the intervention, 31 (86.1%) received the self-guided online intervention plus chat assistance (SGOI+C), whereas five participants (13.9%) received it without chat support (SGOI).

16) For each group, number of participants (denominator) included in each analysis and whether the analysis was by original assigned groups

16-i) Report multiple "denominators" and provide definitions

Report multiple "denominators" and provide definitions: Report N's (and effect sizes) "across a range of study participation [and use] thresholds" [1], e.g., N exposed, N consented, N used more than x times, N used more than y weeks, N participants "used" the intervention/comparator at specific pre-defined time points of interest (in absolute and relative numbers per group). Always clearly define "use" of the intervention.

	1	2	3	4	5	
subitem not at all important	○	○	◉	○	○	essential

Clear selection

Your response is too large. Try shortening some answers.

Does your paper address subitem 16-i? *

Copy and paste relevant sections from the manuscript (include quotes in quotation marks "like this" to indicate direct quotes from your manuscript), or elaborate on this item by providing additional information not in the ms, or briefly explain why the item is not applicable/relevant for your study

aAt the start of the intervention, the main modules were where the participants dropped out. Table 2 presents this information in detail.

16-ii) Primary analysis should be intent-to-treat

Primary analysis should be intent-to-treat, secondary analyses could include comparing only "users", with the appropriate caveats that this is no longer a randomized sample (see 18-i).

	1	2	3	4	5	
subitem not at all important	○	○	○	◉	○	essential

Clear selection

Does your paper address subitem 16-ii?

Copy and paste relevant sections from the manuscript (include quotes in quotation marks "like this" to indicate direct quotes from your manuscript), or elaborate on this item by providing additional information not in the ms, or briefly explain why the item is not applicable/relevant for your study

Our primary hypothesis was to test whether the self-administered intervention with psychological assistance via chat would show more significant statistical gains in reducing anxiety and depression symptoms. For the SGOI group, symptom levels decreased compared to pre and post-test. In contrast, for the SGOI+C group, the reduction in symptoms remained statistically relevant with minor to medium-sized effects for depression, widespread fear, and state anxiety, except for trait anxiety (Table 5).

17a) For each primary and secondary outcome, results for each group, and the estimated effect size and its precision (such as 95% confidence interval)

Your response is too large. Try shortening some answers.

Does your paper address CONSORT subitem 17a? *

Copy and paste relevant sections from the manuscript (include quotes in quotation marks "like this" to indicate direct quotes from your manuscript), or elaborate on this item by providing additional information not in the ms, or briefly explain why the item is not applicable/relevant for your study

3.3 Changes by comparing intervention groups
Our primary hypothesis was to test whether the self-administered intervention with psychological assistance via chat would show more significant statistical gains in reducing anxiety and depression symptoms. For the SGOI group, symptom levels decreased compared to pre and post-test. In contrast, for the SGOI+C group, the reduction in symptoms remained statistically relevant with minor to medium-sized effects for depression, widespread fear, and state anxiety, except for trait anxiety (Table 5).
Post-traumatic symptoms, sleep disturbances, and suicidal ideation showed a reduction after the intervention for the entire group and the two groups separately. However, this decrease in symptomatology did not reach statistically significant levels.

3.5 Follow-up assessment
The follow-up assessment was unavailable for the SGOI group as they did not complete the 3-month or 6-month follow-ups. As for the participants in the SGOI+C group, although overall decreases were observed in the BDI-II and STAI's State Anxiety, they lack statistical relevance. The Widespread Fear Scale maintained a statistically significant decrease at the 3-month follow-up (Rosenthal's R = 0.63). However, the decrease was not maintained in the 6-month evaluation (Table 8).

17a-i) Presentation of process outcomes such as metrics of use and intensity of use

In addition to primary/secondary (clinical) outcomes, the presentation of process outcomes such as metrics of use and intensity of use (dose, exposure) and their operational definitions is critical. This does not only refer to metrics of attrition (13-b) (often a binary variable), but also to more continuous exposure metrics such as "average session length". These must be accompanied by a technical description how a metric like a "session" is defined (e.g., timeout after idle time) [1] (report under item 6a).

	1	2	3	4	5	
subitem not at all important	○	○	◉	○	○	essential

Clear selection

Your response is too large. Try shortening some answers.

Does your paper address subitem 17a-i?

Copy and paste relevant sections from the manuscript (include quotes in quotation marks "like this" to indicate direct quotes from your manuscript), or elaborate on this item by providing additional information not in the ms, or briefly explain why the item is not applicable/relevant for your study

Your answer

17b) For binary outcomes, presentation of both absolute and relative effect sizes is recommended

Does your paper address CONSORT subitem 17b? *

Copy and paste relevant sections from the manuscript (include quotes in quotation marks "like this" to indicate direct quotes from your manuscript), or elaborate on this item by providing additional information not in the ms, or briefly explain why the item is not applicable/relevant for your study

In this manuscript no absolute and relative effect sizes were presented

18) Results of any other analyses performed, including subgroup analyses and adjusted analyses, distinguishing pre-specified from exploratory

Your response is too large. Try shortening some answers.

Does your paper address CONSORT subitem 18? *

Copy and paste relevant sections from the manuscript (include quotes in quotation marks "like this" to indicate direct quotes from your manuscript), or elaborate on this item by providing additional information not in the ms, or briefly explain why the item is not applicable/relevant for your study

3.4 Moderating variables
We hypothesized that coping strategies, acceptance, and satisfaction would function as moderating variables of clinical change in both intervention groups. Due to the low sample size of the SGOI group, moderating analyses were carried out for the whole sample. A series of moderating models were tested. Pre- and post-intervention scores of variables with confirmed changes over intervention (BDI-II, Widespread Fear Scale, and State Anxiety through STAI) were introduced in the models as independent and dependent variables, respectively. Total scores of The Urban Insecurity Scale, System Usability Scale, and Opinion Questionnaire about Treatment were introduced. As we failed to find the moderating effects in these models, we tested a second series of moderating regression for the BDI-II score, using individual items of the Opinion Questionnaire about Treatment (Table 6) and the System Usability Scale (Table 7).

3.6 Acceptance and satisfaction
Among our fourth hypothesis, we tested whether the participants in the SGOI+C group reported higher rates of acceptance and satisfaction compared to the SGOI group. There were no differences regarding satisfaction (Table 6). However, the SGOI+C group scored more positively than the other group's two items on the System Usability Scale. Those items addressed autonomy when using the system (item 4) and confidence when using the system (item 9). No other differences emerged (see Table 7).

18-i) Subgroup analysis of comparing only users

A subgroup analysis of comparing only users is not uncommon in ehealth trials, but if done, it must be stressed that this is a self-selected sample and no longer an unbiased sample from a randomized trial (see 16-iii).

	1	2	3	4	5	
subitem not at all important	○	○	●	○	○	essential

Clear selection

Your response is too large. Try shortening some answers.

Does your paper address subitem 18-i?

Copy and paste relevant sections from the manuscript (include quotes in quotation marks "like this" to indicate direct quotes from your manuscript), or elaborate on this item by providing additional information not in the ms, or briefly explain why the item is not applicable/relevant for your study

Your answer

19) All important harms or unintended effects in each group
(for specific guidance see CONSORT for harms)

Does your paper address CONSORT subitem 19? *

Copy and paste relevant sections from the manuscript (include quotes in quotation marks "like this" to indicate direct quotes from your manuscript), or elaborate on this item by providing additional information not in the ms, or briefly explain why the item is not applicable/relevant for your study

In this manuscript no informatoin about harms or unintended effects are presented

19-i) Include privacy breaches, technical problems

Include privacy breaches, technical problems. This does not only include physical “harm” to participants, but also incidents such as perceived or real privacy breaches [1], technical problems, and other unexpected/unintended incidents. “Unintended effects” also includes unintended positive effects [2].

	1	2	3	4	5	
subitem not at all important	○	○	◉	○	○	essential

Clear selection

Your response is too large. Try shortening some answers.

Does your paper address subitem 19-i?

Copy and paste relevant sections from the manuscript (include quotes in quotation marks "like this" to indicate direct quotes from your manuscript), or elaborate on this item by providing additional information not in the ms, or briefly explain why the item is not applicable/relevant for your study

Due to a technical problem with the platform, at the beginning of the study, the users had only access to the intervention with chat, leaving this a higher weight on that group than the group without chat support. Once identified, it was corrected, and the distribution was ensured. The technical error of the system affected the difference in sample size in the intervention groups. However, this did not affect the equivalence between the groups in terms of pre-assessment indicators (e.g. level of anxiety, depression, etc.).

19-ii) Include qualitative feedback from participants or observations from staff/researchers

Include qualitative feedback from participants or observations from staff/researchers, if available, on strengths and shortcomings of the application, especially if they point to unintended/unexpected effects or uses. This includes (if available) reasons for why people did or did not use the application as intended by the developers.

	1	2	3	4	5	
subitem not at all important	○	○	◉	○	○	essential

Clear selection

Does your paper address subitem 19-ii?

Copy and paste relevant sections from the manuscript (include quotes in quotation marks "like this" to indicate direct quotes from your manuscript), or elaborate on this item by providing additional information not in the ms, or briefly explain why the item is not applicable/relevant for your study

Your answer

DISCUSSION

Your response is too large. Try shortening some answers.

22) Interpretation consistent with results, balancing benefits and harms, and considering other relevant evidence

NPT: In addition, take into account the choice of the comparator, lack of or partial blinding, and unequal expertise of care providers or centers in each group

22-i) Restate study questions and summarize the answers suggested by the data, starting with primary outcomes and process outcomes (use)

Restate study questions and summarize the answers suggested by the data, starting with primary outcomes and process outcomes (use).

	1	2	3	4	5	
subitem not at all important	○	○	○	◉	○	essential

Clear selection

Does your paper address subitem 22-i? *

Copy and paste relevant sections from the manuscript (include quotes in quotation marks "like this" to indicate direct quotes from your manuscript), or elaborate on this item by providing additional information not in the ms, or briefly explain why the item is not applicable/relevant for your study

This study had four objectives. First, to assess the efficacy of a self-guided online intervention (SGOI versus SGOI+C) to reduce clinical symptoms of anxiety and depression in Mexican adults during the first months of the pandemic. Second, to identify the efficacy of the intervention related to the presence or absence of chat support. Third, to test the hypothesized moderating role of coping strategies, usability, and opinion about treatment on clinical change. Fourth, the acceptance and satisfaction of the participants who received the intervention and support through chat were compared to those who received it without chat support

Your response is too large. Try shortening some answers.

22-ii) Highlight unanswered new questions, suggest future research

Highlight unanswered new questions, suggest future research.

	1	2	3	4	5	
subitem not at all important	○	○	○	◉	○	essential

Clear selection

Does your paper address subitem 22-ii?

Copy and paste relevant sections from the manuscript (include quotes in quotation marks "like this" to indicate direct quotes from your manuscript), or elaborate on this item by providing additional information not in the ms, or briefly explain why the item is not applicable/relevant for your study

Future research can investigate the possible factors involved in the lack of long-term effects. Another strength of this study is the inclusion of variables considered mental health stressors, thus expanding from only psychopathological aspects [78]. Although we did not find any statistically significant associations between sleep quality and depression or anxiety, in a recent study by Coiro and colleagues [83], not only did participants report high rates of anxiety and depression during the COVID-19 pandemic, but they also reported poor sleep quality. The authors found a statistically significant association ($p<0.05$) between COVID-19-related stressors (namely sleep quality),depression and anxiety. Therefore, sleep quality may predict mental health situations [84]. Thus, including a sleep quality assessment and coping strategies as moderators of change could explain the phenomenon.

20) Trial limitations, addressing sources of potential bias, imprecision, and, if relevant, multiplicity of analyses

Your response is too large. Try shortening some answers.

20-i) Typical limitations in ehealth trials

Typical limitations in ehealth trials: Participants in ehealth trials are rarely blinded. Ehealth trials often look at a multiplicity of outcomes, increasing risk for a Type I error. Discuss biases due to non-use of the intervention/usability issues, biases through informed consent procedures, unexpected events.

	1	2	3	4	5	
subitem not at all important	○	○	○	◉	○	essential

Clear selection

Your response is too large. Try shortening some answers.

Does your paper address subitem 20-i? *

Copy and paste relevant sections from the manuscript (include quotes in quotation marks "like this" to indicate direct quotes from your manuscript), or elaborate on this item by providing additional information not in the ms, or briefly explain why the item is not applicable/relevant for your study

Your response is too large. Try shortening some answers.

6. Limitations and future challenges
The study has several limitations and has identified relevant elements that can be prevented or addressed to improve future similar projects. The main limitation is the small sample size for the SG-OI group. Therefore, the planned analysis was modified to evaluate the second hypothesis regarding the moderating role of coping strategies, acceptance, and satisfaction variables on clinical change by group. Initially, this analysis was intended to be done separately for each group (SG-OI and SG-OI+C) , but instead, it was performed considering the total sample. Furthermore, it was not possible to assess whether the changes were maintained for the SG OI group, as none completed follow-up at 3 or 6 months.
The inclusion of a therapist should be considered for future applications, as this feature has been reported to generate better results in Internet-based psychological interventions than self-administered ones [77, 86]. However, cost-benefit should be evaluated. As shown by Ruwaard et al [87] a decade of studying internet-based treatments conducted in nine randomized controlled clinical trials, online therapist-assisted CBT has provided evidence supporting its effectiveness and efficacy of its application with outcomes comparable to in-person clinical practices, including high adherence rates.
A further limitation is the sample characteristics, which only comprise participants who accessed the Mental Health COVID-19 platform seeking treatment. This could lead to biases, as the group included people with access to technology who were actively seeking psychological help. This situation prevents generalizing the findings to the Mexican population, as it is a non-probabilistic convenience sampling. Another limitation was the significant difference in depressive symptoms at baseline between the participants of both groups, which prevented adequate comparison before entering the study conditions. Caution should be taken in conclusions about this clinical variable. In subsequent studies, it would be relevant to assess the potential repercussions of this condition regarding treatment evolution, adherence, and dropout rates.
There was another possible bias in the sample. There were more women compared to men (80.6% versus 19.4%) and more participants with university studies (77.8%) compared to other lower educational levels. Other online intervention studies have shown similar characteristics regarding gender and educational attainment [88], highlighting the importance of developing future treatments aimed at men and people with lower educational backgrounds. Furthermore, it is advisable to explore strategies such as establishing collaborations with public centers or corporations to facilitate access to this population group.

Another study limitation is the small sample size of the people who completed the intervention (n=36). Future studies should increase the number of users, for example, reviewing the recruitment criteria and interviewing potential participants to explore dropout reasons, barriers, and possible stigmas surrounding mental health. Online interventions often face difficulties such as medium to high dropout rates [88] and low adherence to treatment [89]. In this study, it was observed that the group that had chat support was able to complete the follow-up at 6 months, compared to those without therapist guidance. Having contact with a professional could be an element that helps adherence to the program. Conversely, in the satisfaction evaluation, items related to the system's complexity and the intervention's utility were associated with worse treatment outcomes. These elements could have contributed to the lack of adherence in some participants. Implementing pilot tests of the platforms in the general population to detect such elements would facilitate the success of the online intervention. Furthermore, this [illegible]

Your response is too large. Try shortening some answers.

[illegible] validations carried out with the target population. These instruments with updated

[illegible] psychometric properties will reduce possible biases related to the measurement periods. Moreover, in low- and middle-income countries, although mental health care is needed, it is not easily available. Online interventions could help reduce this gap [90]. For instance, Wang et al [91] found that self-help Web-based programs can aid people who have experienced traumatic events. The participants in their study those who belonged to the rural group did not have easy Internet access, were supported with Web use throughout the process. This resulted in a positive adherence to the program. However, the authors speculate that their results could have been related to the face-to-face contact between the participants and the volunteers who helped with the Internet service problems. Furthermore, a study conducted by Benjet et al. (92) in Colombia and Mexico with a large sample size of 1,319 university students, comparing i-CBT, self-guided i-CBT and Treatment as usual, obtaining significant reductions in anxiety and depression.

In addition, another situation to consider is that online psychological interventions - particularly those of a self-guided type- are still relatively unknown in Latin American countries because these interventions are often offered to the population as therapeutic alternatives for a short time. Furthermore, few online interventions have been delivered in Mexico, free of charge for the user (for instance, Grief COVID; [93]. Privacy and security of the users' data should be explicitly warranted to increase trust among potential users of online resources [94]. These circumstances could have influenced the low participation that was evidenced in this study, and they should be considered in the elaboration of future online interventions.

An additional limitation is the small number of participants who completed the intervention in the SG OI group (n=5) compared to those from the SG OI+C group (n=31). This situation prevented comparing the intervention's effectiveness in each group separately, which would have contributed to the study. Nevertheless, these results emphasize the importance of including elements like chat in self-guided online interventions that establish more direct and personalized contact with users. This interaction element was probably crucial to achieving greater adherence to treatment. Other studies that have used chat [95] as a tool for online psychologically guided interventions have shown that these resources have promoted the permanence of users and have been positively valued by them. The results of this study also show this trend -identified in several of the works mentioned above highlighting the role of interactive elements such as chat, which act as resources that could encourage permanence in the intervention. Fifty-six percent of the participants did not complete the intervention, 16% more than estimated in the sample size calculation, which represents a high rate of dropouts. However, this rate is reported in the literature on web-based treatments [53, 54] and could be explained by the same condition of emotional discomfort of the participants, the lack of private and adequate space for carrying out their intervention in the context of confinement due to the COVID-19 pandemic. Future analysis could be conducted with the current data collected to analyze the variables that could predict a higher likelihood of dropping out, such as the use of machine learning, a tool increasingly used in diverse studies to predict dropout [96] or completion [97] in online interventions, among other uses.
Another limitation of the study was the computer errors faced during the study. Regarding the randomization error, the platform utilized Tawk.io, which is a chat software designed to create live communication between users and the applications team, at first the basic integration was built over the web on the client side, but this brought the problem of not being able to determine the user who was logged into the platform meaning the [illegible]

Your response is too large. Try shortening some answers.

[illegible] implementation of the Tawk.io plugin was transferred to server-side code, in this context we

do have a clear identification of user logged and correct assignment of the chat classification.
Furthermore, the Positive Psychological Functioning scale [98] was planned to be included in this article. However, it could not be included because while the post-measurements could be retrieved, there were electronic issues during the pre-measurement.
Finally, psychotic disorder was proposed as an exclusion criterion in the study protocol. Nevertheless, we did not use this criterion when we conducted the study. We mention here this inconsistency as a limitation of this research. Further interventions aimed at the general population should explore the presence of psychotic symptoms.

21) Generalisability (external validity, applicability) of the trial findings

NPT: External validity of the trial findings according to the intervention, comparators, patients, and care providers or centers involved in the trial

21-i) Generalizability to other populations

Generalizability to other populations: In particular, discuss generalizability to a general Internet population, outside of a RCT setting, and general patient population, including applicability of the study results for other organizations

	1	2	3	4	5	
subitem not at all important	○	○	○	◉	○	essential

Clear selection

Does your paper address subitem 21-i?

Copy and paste relevant sections from the manuscript (include quotes in quotation marks "like this" to indicate direct quotes from your manuscript), or elaborate on this item by providing additional information not in the ms, or briefly explain why the item is not applicable/relevant for your study

Your answer

Your response is too large. Try shortening some answers.

21-ii) Discuss if there were elements in the RCT that would be different in a routine application setting

Discuss if there were elements in the RCT that would be different in a routine application setting (e.g., prompts/reminders, more human involvement, training sessions or other co-interventions) and what impact the omission of these elements could have on use, adoption, or outcomes if the intervention is applied outside of a RCT setting.

	1	2	3	4	5	
subitem not at all important	○	○	○	◉	○	essential

Clear selection

Does your paper address subitem 21-ii?

Copy and paste relevant sections from the manuscript (include quotes in quotation marks "like this" to indicate direct quotes from your manuscript), or elaborate on this item by providing additional information not in the ms, or briefly explain why the item is not applicable/relevant for your study

Your answer

OTHER INFORMATION

23) Registration number and name of trial registry

Does your paper address CONSORT subitem 23? *

Copy and paste relevant sections from the manuscript (include quotes in quotation marks "like this" to indicate direct quotes from your manuscript), or elaborate on this item by providing additional information not in the ms, or briefly explain why the item is not applicable/relevant for your study

Trial Registration: NCT04468893, and in the International Registered Report Identifier (IRRID): DERR1-10.2196/23117

Your response is too large. Try shortening some answers.

24) Where the full trial protocol can be accessed, if available

Does your paper address CONSORT subitem 24? *

Cite a Multimedia Appendix, other reference, or copy and paste relevant sections from the manuscript (include quotes in quotation marks "like this" to indicate direct quotes from your manuscript), or elaborate on this item by providing additional information not in the ms, or briefly explain why the item is not applicable/relevant for your study

Further study details are available in the protocol manuscript by Dominguez-Rodriguez et al., [49].

49 . Dominguez-Rodriguez A, De La Rosa-Gómez A, Hernández Jiménez MJ et al. A self-administered multicomponent web-based mental health intervention for the mexican population during the COVID-19 pandemic: protocol for a randomized controlled trial. JMIR Res Protoc. 2020; 9(11):e23117. doi:10.2196/23117

25) Sources of funding and other support (such as supply of drugs), role of funders

Does your paper address CONSORT subitem 25? *

Copy and paste relevant sections from the manuscript (include quotes in quotation marks "like this" to indicate direct quotes from your manuscript), or elaborate on this item by providing additional information not in the ms, or briefly explain why the item is not applicable/relevant for your study

Funding
The Tecnológico de Monterrey, México, supported the publication fees of this manuscript. The funding body had no role in the study design, manuscript writing, or submitting the paper to a specific journal.

X27) Conflicts of Interest (not a CONSORT item)

Your response is too large. Try shortening some answers.

X27-i) State the relation of the study team towards the system being evaluated

In addition to the usual declaration of interests (financial or otherwise), also state the relation of the study team towards the system being evaluated, i.e., state if the authors/evaluators are distinct from or identical with the developers/sponsors of the intervention.

	1	2	3	4	5	
subitem not at all important	○	○	◉	○	○	essential

Clear selection

Does your paper address subitem X27-i?

Copy and paste relevant sections from the manuscript (include quotes in quotation marks "like this" to indicate direct quotes from your manuscript), or elaborate on this item by providing additional information not in the ms, or briefly explain why the item is not applicable/relevant for your study

Conflict of Interest
The authors declare that the research was conducted in the absence of any commercial or financial relationships that could be construed as a potential conflict of interest.

About the CONSORT EHEALTH checklist

As a result of using this checklist, did you make changes in your manuscript? *

- ○ yes, major changes
- ○ yes, minor changes
- ◉ no

Your response is too large. Try shortening some answers.

What were the most important changes you made as a result of using this checklist?

Only adding the developer of the intervention

How much time did you spend on going through the checklist INCLUDING making changes in your manuscript *

Around 2 hours including the changes in the manuscript.

As a result of using this checklist, do you think your manuscript has improved? *

- (x) yes
- ( ) no
- ( ) Other:

Would you like to become involved in the CONSORT EHEALTH group?

This would involve for example becoming involved in participating in a workshop and writing an "Explanation and Elaboration" document

- ( ) yes
- (x) no
- ( ) Other:

Clear selection

Any other comments or questions on CONSORT EHEALTH

Your answer

Your response is too large. Try shortening some answers.

STOP - Save this form as PDF before you click submit

To generate a record that you filled in this form, we recommend to generate a PDF of this page (on a Mac, simply select "print" and then select "print as PDF") before you submit it.

When you submit your (revised) paper to JMIR, please upload the PDF as supplementary file.

Don't worry if some text in the textboxes is cut off, as we still have the complete information in our database. Thank you!

Final step: Click submit !

Click submit so we have your answers in our database!

Submit Clear form

Never submit passwords through Google Forms.

This content is neither created nor endorsed by Google. Report Abuse - Terms of Service - Privacy Policy

Google Forms

Your response is too large. Try shortening some answers.

Your response is too large. Try shortening some answers.
